# Interpretable machine-learning risk stratification at the time of diagnosis for 3-year mortality in *de novo* metastatic prostate cancer: development and temporal validation in SEER

**DOI:** 10.3389/fonc.2026.1904589

**Published:** 2026-08-31

**Authors:** Xin Wang, Guanglei Yao, Heqian Liu, Wei Ding

**Affiliations:** Department of Urology, The Second People’s Hospital of Wuhu, Wuhu, Anhui, China

**Keywords:** cancer-specific mortality, CatBoost, explainable artificial intelligence, machine learning, metastatic prostate cancer, prognostic model, risk stratification, SEER

## Abstract

Men with *de novo* metastatic prostate cancer (mPCa) face a poor but heterogeneous prognosis, yet interpretable, transparently reported diagnostic-time tools remain limited. Using the Surveillance, Epidemiology, and End Results (SEER) 17-registry database (2010–2020), we developed and temporally validated machine-learning models to predict fixed 3-year cancer-specific mortality (CSM) and overall mortality (OM) in newly diagnosed mPCa. From 42,691 exported cases, we defined a cohort of 41,057 (survival ≥1 month) and split it temporally into training (2010–2016, *n* = 21,584) and validation (2017–2020, *n* = 19,473) sets. Seven algorithms were benchmarked on an identical diagnostic-time feature space [age, year, race, marital status, prostate-specific antigen (PSA), Gleason, and metastatic sites], excluding treatment and American Joint Committee on Cancer (AJCC) T/N; CatBoost was selected as the primary model. In temporal validation, it achieved an area under the curve (AUC) of 0.701 [95% confidence interval (CI) 0.693–0.708] for net CSM, a cause-specific quantity excluding other-cause deaths, not an absolute competing-risks cumulative incidence, and 0.704 (0.697–0.712) for OM, with good calibration (slopes 1.12 and 1.08) and positive decision-curve net benefit for flagging high-risk patients across roughly 30%–70% thresholds; discrimination was comparable across leading algorithms and to logistic regression. Temporal internal–external cross-validation confirmed stable discrimination (pooled AUC 0.701/0.703; 95% prediction intervals from 0.672 to 0.725); because diagnosis year is a model feature, this establishes stability within 2010–2020, and the model should not be applied to later diagnosis years without local recalibration. Full validation-set SHapley Additive exPlanations (SHAP) analysis identified age, Gleason score, PSA, diagnosis year, and metastatic sites as dominant predictors, and training-derived risk tertiles separated validation event rates monotonically (net CSM 28%/49%/67%; OM 34%/57%/74%). Transportability was limited: in an independent non-SEER single-center cohort (*n* = 150), discrimination was attenuated (cancer-specific AUC 0.59, 95% CI 0.49–0.69; overall 0.59, 0.50–0.68) and calibration drifted markedly [slope 0.47; observed-to-expected ratio (O:E) 1.23 and 1.19], with only the OM risk ordering preserved as a monotone gradient. This interpretable model therefore provides calibrated diagnostic-time risk stratification within SEER 2010–2020, intended not for treatment selection but for prognostic counseling, closer follow-up, and trial enrichment; local intercept-and-slope recalibration and larger multicenter validation are required before use outside that setting or window.

## 1 Introduction

Metastatic prostate cancer at presentation (*de novo* mPCa) is almost always ultimately lethal, yet its course is strikingly heterogeneous: some men die within a year of diagnosis while others survive many years (1, 2). This heterogeneity matters at the very first encounter. Care has shifted decisively toward upfront intensification—adding docetaxel and/or an androgen-receptor pathway inhibitor to androgen-deprivation therapy (3–11)—which improves survival but widens the outcome spread and makes decisions about how aggressively to treat, and whom to enrol in trials, increasingly risk-dependent (12–14). In this setting, clinicians and patients need an individualized 3-year overall mortality (OM) estimate, together with net cancer-specific risk stratification, at the time of diagnosis—before treatment is chosen—to support honest prognostic counseling, to calibrate the intensity of follow-up, and to enrich clinical trials with the high-risk patients most likely to benefit from intensification. Such an estimate must rest only on information available at diagnosis.

Existing prognostic instruments for mPCa are predominantly traditional nomograms or single-algorithm models (15, 16), and machine-learning (ML) approaches are often criticized as opaque “black boxes” and for lacking transparent reporting and calibration (17, 18). Two questions therefore remain practically important: whether a rigorously developed, interpretable ML model adds value as a diagnostic-time risk tool, and exactly where its information is, and is not, useful.

Here, we develop and temporally validate interpretable ML models for fixed 3-year CSM and OM in a large Surveillance, Epidemiology, and End Results (SEER) cohort of *de novo* mPCa. We benchmark seven algorithms under a unified pipeline, select a primary CatBoost model, and interpret it with full validation-set SHapley Additive exPlanations (SHAP). We deliberately bound the claims: we report calibration and decision-curve utility alongside discrimination; we treat treatment-associated analyses as observational and hypothesis-generating, noting that SEER cannot capture the androgen-deprivation and androgen-receptor pathway therapies that dominate modern mPCa care; and we frame the model as a tool for risk stratification and communication, not for treatment selection.

## 2 Materials and methods

This study was developed and is reported following the TRIPOD+AI statement (19) (completed checklist in **Supplementary Table 8**), with risk of bias and applicability considerations informed by PROBAST+AI (20). The SEER development and temporal validation analyses used only de-identified, publicly available secondary data and were exempt from institutional review board review; the independent external validation cohort (Section 2.7) comprised retrospectively collected, de-identified single-center records, used with the approval of the Ethics Committee of The Second People’s Hospital of Wuhu (approval number 2024-KY-158) and a waiver of individual informed consent, in accordance with the Declaration of Helsinki (see the Ethics statement).

### 2.1 Data source and cohort

Data were obtained from the SEER Research Data, 17 Registries, November 2025 submission (21). In SEER*Stat, we selected cases of malignant prostate cancer (primary site C61.9) with distant disease at diagnosis—defined by the Combined Summary Stage with Expanded Regional Codes (2004+) category “Distant site(s)/node(s) involved”—diagnosed 2010–2020, restricted to malignant behavior and known age; histology was not further restricted, so the cohort comprises malignant prostate tumors of all histologies (overwhelmingly adenocarcinoma). The full case-selection statement and the analysis variable dictionary are provided in the **Supplementary Methods** and **Supplementary Table 9**, and we applied the analytic exclusions below. From 42,691 exported cases, we excluded 1,634 with survival shorter than 1 month (1,606 with <1 month and 28 with missing survival time) to avoid unstable or ambiguous diagnosis-month records, yielding a main analytic cohort of 41,057. Retention of 0-month cases was assessed in a sensitivity analysis (Section 2.10).

### 2.2 Temporal validation design

The cohort was partitioned by year of diagnosis into a training set (2010–2016, *n* = 21,584) and a temporally independent validation set (2017–2020, *n* = 19,473). This is an internal temporal validation and is not described as external validation.

### 2.3 Endpoints and the fixed-horizon evaluability rule

Two prespecified fixed-horizon endpoints were studied: 3-year cancer-specific mortality (CSM; cancer-specific death by 36 months) and 3-year OM, each defined as a binary classification target at the 36-month horizon. A 3-year (36-month) horizon was chosen *a priori* as a clinically meaningful medium-term landmark in *de novo* metastatic disease: it lies beyond the early high-mortality phase and captures sustained rather than only short-term risk (unlike a 1-year endpoint), while remaining far enough inside the available follow-up to limit censoring and to reduce sensitivity to therapy-era drift and incomplete long-term follow-up that would affect a 5-year endpoint in a 2010–2020 cohort. A patient was evaluable if the 36-month status was determinate—that is, follow-up reached 36 months or the relevant death occurred by 36 months—and the outcome was positive if the relevant death occurred at or before 36 months. For OM, patients alive but censored before 36 months were not evaluable. For CSM, both patients censored before 36 months and patients who died of other (non-cancer) causes before 36 months were treated as non-evaluable. The primary CSM target is therefore a complete-case, cause-specific (“net”) probability of cancer death by 36 months among patients with determinate status, not a competing-risks cumulative incidence: this estimand is transparent and matches the diagnostic-time classification setting, but because it removes other-cause deaths, it does not estimate the absolute cumulative incidence of cancer death in the presence of competing mortality. The competing-risks perspective is addressed directly by a Fine–Gray subdistribution sensitivity analysis (Section 2.9; **Supplementary Table 5**), against which the primary model was checked for concordance. Applying the evaluability rule to the validation cohort yielded 19,122 evaluable for OM (10,048 deaths) and 16,806 evaluable for CSM (7,690 cancer deaths). The OM exclusion was small (351/19,473, 1.8%), indicating that temporal validation was not materially shortened by incomplete follow-up; the larger CSM exclusion reflected competing other-cause mortality rather than loss to follow-up. Cause of death used the SEER cause-specific death classification, which may be subject to misclassification. We retained a fixed-horizon binary classifier as the primary model rather than a landmark competing-risks survival model for three reasons. First, the decision the tool supports is itself fixed-horizon and binary—whether to flag a newly diagnosed patient as high risk over the next 3 years—so the classifier matches the deployment target directly. Second, the cost of the evaluability rule is small and quantified: only 1.8% of the validation cohort (351/19,473) was excluded for OM, and an inverse-probability-of-censoring-weighted analysis on the entire cohort reproduced the complete-case result [area under the curve (AUC) 0.703 versus 0.704; **Supplementary Table 10**]. Third, the survival alternatives were fitted and did not outperform the classifier [Cox 3-year AUC 0.698; cause-specific Cox 0.694; Fine–Gray 0.676; random survival forest 0.700 (overall) and 0.695 (cancer-specific); **Supplementary Table 5**]. We nevertheless acknowledge the genuine trade-off: a landmark competing-risks model targets the absolute cumulative incidence, which is the quantity most directly interpretable at the bedside. We therefore report the absolute competing-risks cumulative incidence by risk group as a companion analysis (**Supplementary Table 26**), and note that the OM endpoint is itself an absolute, all-cause quantity.

### 2.4 Predictors

The diagnostic-time model used only variables available at or near diagnosis: age (SEER age-group midpoint), year of diagnosis (continuous), race, marital status, prostate-specific antigen (PSA) (SEER 2010+ lab-value recode), Gleason score (SEER 2010+ clinical recode), and metastatic sites (bone, liver, lung, and brain). Treatment variables and AJCC T/N stage were excluded from the primary model by design rather than by omission. Treatment is a post-diagnosis decision: including it in a diagnostic-time predictor set would introduce information leakage and confound a tool intended to inform—not to follow—treatment choices. Derived AJCC T/N stage was populated only for 2010–2015 in this export, so including it would have broken the temporal validation design by making the predictor set inconsistent between the 2010–2016 training and 2017–2020 validation periods; the metastatic-disease definition therefore used Combined Summary Stage, which is available across all study years. Unknown/missing values were retained as explicit categories to preserve sample size and reflect real-world information availability; categorical predictors were one-hot encoded.

### 2.5 Model development and primary-model selection

Seven algorithms were trained on an identical one-hot feature space under a unified pipeline: logistic regression, elastic net, random forest, XGBoost (22), LightGBM (23), CatBoost (24), and a Platt-calibrated linear support-vector machine. Hyperparameters were selected by a 20-candidate, fivefold cross-validation within the training cohort. No formal *a priori* sample-size calculation was performed, as the development cohort size was fixed by the SEER extraction; with 21,584 training patients and several thousand 3-year events per endpoint relative to 35 model columns after one-hot encoding (well over 100 events per candidate parameter for both CSM and OM), the effective sample size far exceeded the minimums recommended for stable prediction-model development (25). CatBoost was trained on the same one-hot matrix as the other algorithms rather than using native categorical handling, to preserve an identical feature space for fair cross-algorithm comparison under the planned cross-validation framework. Models were judged against a prespecified hierarchy: we first required acceptable calibration (calibration slope near 1 with no systematic miscalibration), then compared discrimination and overall performance (AUC, Brier score, and average precision) and decision-curve net benefit, and only then weighed interpretability and reproducibility. CatBoost met the calibration requirement, had the best or equal-best discrimination and net benefit, and supported exact full-cohort SHAP interpretation; it was therefore selected as the primary model. Absolute differences among the leading algorithms were small, particularly between CatBoost and random forest for CSM, but random forest was less well calibrated (Section 3.3). Endpoint-specific diagnostic-time CatBoost models were trained under a frozen specification (fixed seed, recorded hyperparameters, and final iteration counts; **Supplementary Table 19**); the original primary-model object was not retained, so the models reported here were retrained under that frozen specification and archived for reuse, reproducing the original development predictions almost exactly (Spearman *r* ≈ 0.997).

### 2.6 Performance, interpretation, and risk stratification

In the temporal validation cohort, we assessed discrimination [AUC with DeLong (26) and 1,000× paired stratified bootstrap 95% confidence intervals (CIs)], overall performance (Brier score, average precision), calibration (calibration-in-the-large/intercept, calibration slope, observed-to-expected ratio, observed-versus-predicted risk by decile, and calibration plots), and clinical utility [decision-curve analysis (27)]; the selection and interpretation of these performance measures followed recent guidance for evaluating clinical prediction models that use ML (28). For the primary model, we computed SHAP values (29) over the entire validation cohort (CSM *n* = 16,806; OM *n* = 19,122) without sampling or approximation, verifying the additivity property (maximum deviation <1 × 10^-14^). Validation patients were assigned to low-, intermediate-, and high-risk groups using cut points fixed at the tertiles of training-cohort predicted risk (CSM 0.461/0.595; OM 0.515/0.657), and observed event rates per group were reported. To verify that the fixed-horizon (complete-case) estimands were not distorted by early censoring or, for CSM, by competing other-cause mortality, we additionally evaluated the primary model’s predicted 3-year risks on the entire validation cohort using inverse-probability-of-censoring weighting (IPCW), reporting IPCW time-dependent AUC and IPCW prediction error at 36 months (cancer death as the event of interest with other-cause death as a competing event for CSM; all-cause death for OM).

### 2.7 External validation in an independent cohort

To assess transportability beyond the SEER development setting and to a different healthcare system, the locked primary models were applied—without any refitting or recalibration—to an independent, geographically and ethnically distinct cohort that shares no patients, registry, or coding system with SEER: 150 consecutive men with *de novo* mPCa managed at a single Chinese tertiary center (The Second People’s Hospital of Wuhu; diagnosed 2011–2018). Records were mapped onto the identical 35-feature diagnostic-time space and scored for fixed 3-year CSM and OM risk; the same 36-month evaluability rule and the same frozen, training-derived risk-group cut points (CSM 0.461/0.595; OM 0.515/0.657) were applied. We report discrimination (AUC with DeLong 95% CIs), calibration [slope, calibration-in-the-large intercept, observed-to-expected ratio (O:E), and a flexible recalibration curve], the Brier score, and observed 3-year mortality within the predefined low-, intermediate-, and high-risk groups; as an internal control, the scoring pipeline was confirmed to reproduce the locked SEER validation AUC (0.701/0.704) and risk-group cut points. Because the model’s only Asian race category is the US SEER “Asian or Pacific Islander” code, its application to a mainland-Chinese cohort is itself a transportability assumption; diagnosis years spanned 2011–2018, so patients diagnosed in 2017–2018 lie beyond the 2010–2016 training range of the year-of-diagnosis feature and are extrapolated on that predictor; because year of diagnosis carries calendar-time information about the absolute risk level (**Supplementary Table 25**), this is a further reason to expect calibration drift on transport.

### 2.8 Secondary treatment-associated analyses (observational)

As secondary, exploratory analyses, we described SEER-documented treatment, evaluated whether adding treatment variables improved prediction (treatment-augmented models), and explored risk-stratified treatment–outcome associations using stabilized inverse-probability-of-treatment weighting (IPTW) (30) within risk groups, with covariate-balance and weight diagnostics. These analyses are observational and hypothesis-generating. Importantly, SEER does not capture androgen-deprivation therapy or androgen-receptor pathway inhibitors—the systemic agents central to contemporary metastatic hormone-sensitive disease—and chemotherapy is recorded only as documented “yes” versus “no/unknown”; “no/unknown” therefore cannot be equated with untreated, and no causal interpretation is made.

### 2.9 Survival-modeling sensitivity analyses

As sensitivity analyses, we fitted Cox proportional-hazards (OM), cause-specific Cox and Fine–Gray competing-risk (31) (CSM), and random survival forest (32) models on the same diagnostic-time predictors (full-rank dummy coding), reporting the Harrell C-index, 3-year time-dependent AUC, and Brier score. These models were not intended to replace the primary fixed-time CatBoost model.

### 2.10 Robustness

Prespecified robustness analyses included retention of 0-month survival cases; subgroup performance and calibration within prespecified subgroups (age group, race, PSA known/unknown, Gleason known/unknown, visceral versus non-visceral metastasis, and diagnosis year, with per-subgroup *n*, events, AUC, Brier score, and calibration slope); missing/unknown-category sensitivity comparing the primary missing-indicator approach (unknown values retained as explicit categories) with complete-case analysis and with simple imputation; 3- and 6-month landmark analyses; and strict treatment-coding sensitivity. A registry-level geographic internal–external validation was planned but could not be conducted because the SEER registry identifier is suppressed in the SEER Research Data and Research Limited-Field products available to us and is released only through SEER Research Plus, to which we had no access; the deposited leave-one-registry-out code is runnable should registry-level data become available. Because year of diagnosis is recorded for every patient, we instead performed a fully data-complete temporal internal–external cross-validation. In the primary scheme (leave-one-calendar-year-out), each diagnosis year (2010–2020; 11-fold) was held out once and scored by the frozen primary CatBoost model refit on all other years, so every patient was predicted exactly once by a model blind to its own year; hyperparameters were tuned once on the full development cohort and held fixed (refit, not retune), year of diagnosis was retained as a model feature (so the held-out interior year is interpolated), and the calendar-period label served only as a partition key. Between-year heterogeneity was pooled by DerSimonian–Laird random-effects meta-analysis on the logit-AUC scale, reporting *I*² and a 95% prediction interval. As a secondary, deployment-realistic scheme, a prospective rolling-origin analysis trained only on years before each test year and scored that year. All folds met a prespecified threshold of at least 200 patients and 100 events per held-out year.

### 2.11 Software and reproducibility

Analyses used R 4.4.1 (survival, cmprsk, randomForestSRC, pROC, and ggplot2) and Python (scikit-learn, CatBoost, XGBoost, LightGBM, and SHAP), with a fixed random seed. The analysis code, run scripts, and predictor dictionary are openly available in a public repository (33) (see the Data availability statement).

## 3 Results

### 3.1 Cohort

**Figure 1:**
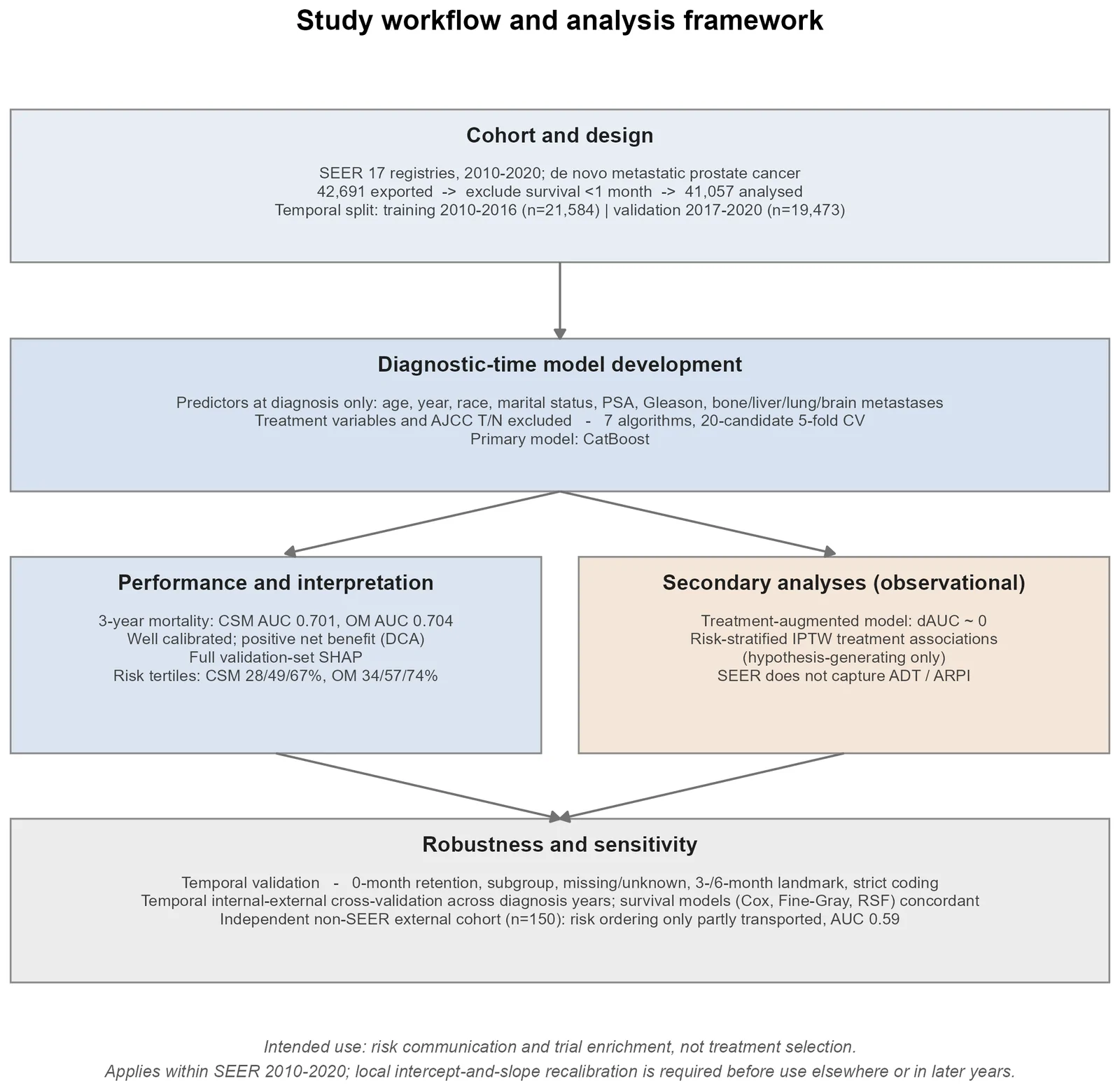
Study workflow and analysis framework. SEER cohort selection (42,691 exported → 41,057 main cohort), temporal split (training 2010–2016, *n* = 21,584; validation 2017–2020, *n* = 19,473), diagnostic-time seven-algorithm modeling with CatBoost selected as the primary model, full validation-set SHAP interpretation, training-tertile risk stratification, and secondary observational treatment analyses, supported by the robustness/sensitivity battery.

The main cohort comprised 41,057 men with *de novo* mPCa (training 21,584; temporal validation 19,473); the study workflow and analysis framework are summarized in **Figure 1**. The cohort was elderly [median age, 72 years; interquartile range (IQR) 62–82] and predominantly White (75.8%). High disease burden was typical: PSA was ≥98 ng/mL (SEER ceiling category) in 46.8% and Gleason was 9–10 in 35.1% (Gleason not assessable/no biopsy in 41.6%). Bone metastases were present in 86.4%, with visceral involvement less common (lung 8.5%, liver 4.2%, and brain 1.0%). Documented treatment comprised radiation in 23.5% and chemotherapy in 15.0%. Baseline characteristics were closely balanced between the training and validation periods (**Table 1**). Over the cohort, 77.0% had died by the study cutoff. Histology was overwhelmingly adenocarcinoma (85.6%); a further 13.0% were carcinoma or malignant neoplasm not otherwise specified, and only 1.5% were specified non-adenocarcinoma variants (small-cell or neuroendocrine carcinoma 1.2%; other 0.3%) (**Supplementary Table 16**).

**Table 1:** Baseline characteristics of the *de novo* mPCa cohort, overall and by temporal split.

Variable	Level	Overall (*N* = 41,057)	Training 2010–2016 (*N* = 21,584)	Validation 2017–2020 (*N* = 19,473)
Age, years	Median [IQR]	72 [62–82]	72 [62–82]	72 [62–82]
Race	White	31,131 (75.8)	16,455 (76.2)	14,676 (75.4)
	Black	6,752 (16.4)	3,551 (16.5)	3,201 (16.4)
	Asian/Pacific Islander	2,652 (6.5)	1,316 (6.1)	1,336 (6.9)
	American Indian/Alaska Native	316 (0.8)	183 (0.8)	133 (0.7)
	Unknown	206 (0.5)	79 (0.4)	127 (0.7)
Marital status	Married	23,224 (56.6)	12,012 (55.7)	11,212 (57.6)
	Unmarried	15,352 (37.4)	8,062 (37.4)	7,290 (37.4)
	Unknown	2,481 (6.0)	1,510 (7.0)	971 (5.0)
PSA, ng/mL	<10	3,574 (8.7)	1,944 (9.0)	1,630 (8.4)
	10–20	3,408 (8.3)	1,740 (8.1)	1,668 (8.6)
	20–50	5,394 (13.1)	2,816 (13.0)	2,578 (13.2)
	50–<98	4,345 (10.6)	2,228 (10.3)	2,117 (10.9)
	≥98 (SEER ceiling)	19,205 (46.8)	10,057 (46.6)	9,148 (47.0)
	Unknown	5,131 (12.5)	2,799 (13.0)	2,332 (12.0)
Gleason score	≤6	478 (1.2)	329 (1.5)	149 (0.8)
	7	3,248 (7.9)	1,917 (8.9)	1,331 (6.8)
	8	5,833 (14.2)	3,213 (14.9)	2,620 (13.5)
	9	11,758 (28.6)	6,053 (28.0)	5,705 (29.3)
	10	2,643 (6.4)	1,382 (6.4)	1,261 (6.5)
	Not assessed/no biopsy	17,097 (41.6)	8,690 (40.3)	8,407 (43.2)
Bone metastasis	Yes	35,484 (86.4)	18,641 (86.4)	16,843 (86.5)
	No	4,853 (11.8)	2,428 (11.2)	2,425 (12.5)
	Unknown	720 (1.8)	515 (2.4)	205 (1.1)
Liver metastasis	Yes	1,731 (4.2)	902 (4.2)	829 (4.3)
Lung metastasis	Yes	3,482 (8.5)	1,667 (7.7)	1,815 (9.3)
Brain metastasis	Yes	430 (1.0)	236 (1.1)	194 (1.0)
Surgery (primary site)	Yes	4,266 (10.4)	2,308 (10.7)	1,958 (10.1)
Radiation	Yes	9,654 (23.5)	4,836 (22.4)	4,818 (24.7)
Chemotherapy	documented Yes	6,166 (15.0)	2,987 (13.8)	3,179 (16.3)
Follow-up, months	Median [IQR]	31 [13–53]	29 [12–62]	33 [14–50]

Cells are *n* (%) unless noted. Visceral-metastasis and treatment rows show the positive category; remaining patients are No/Unknown (full categories in the **Supplementary Materials**). Chemotherapy is SEER “documented yes vs. no/unknown”. The training and validation periods are closely balanced.

### 3.2 Primary model performance

**Figure 2:**
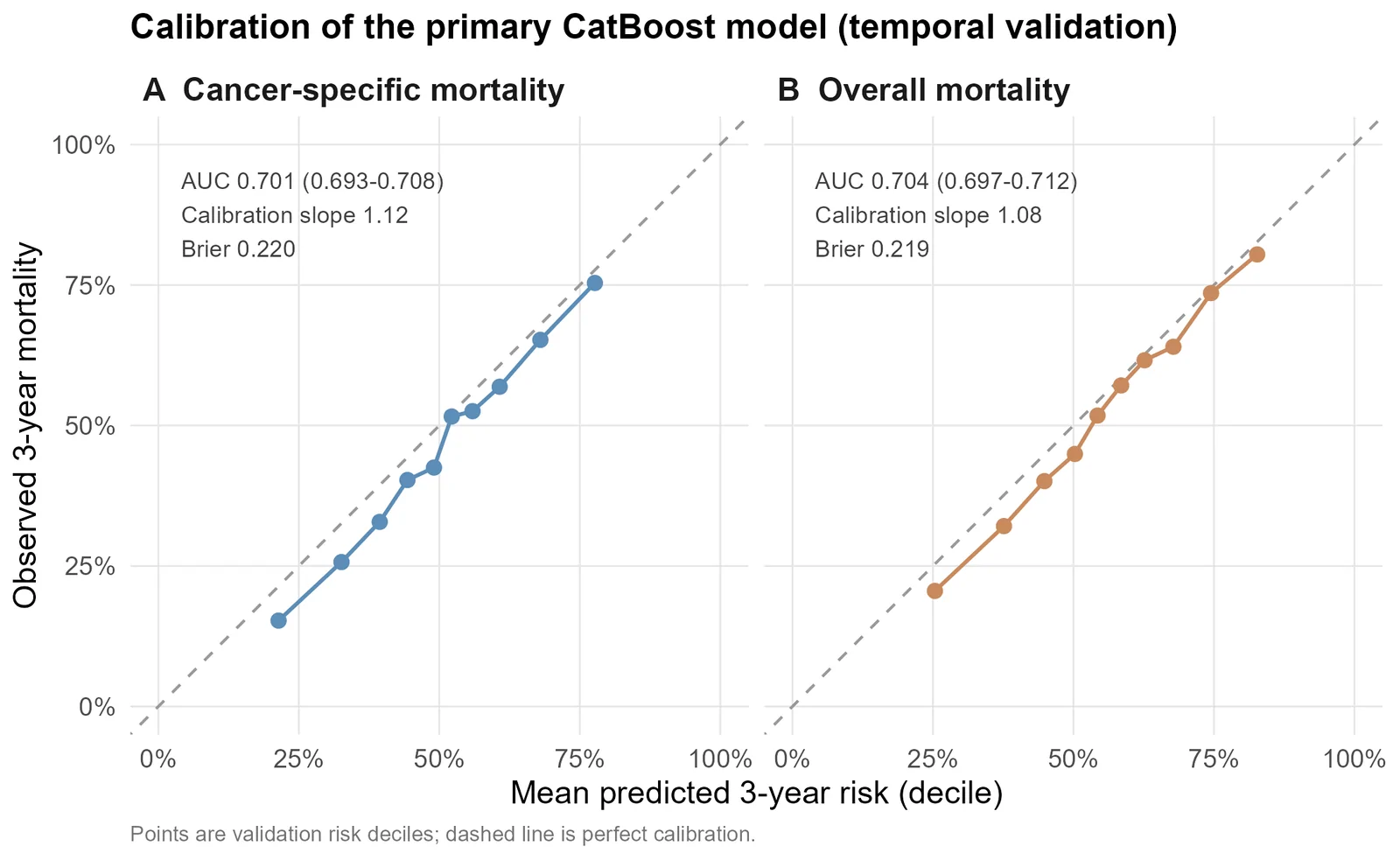
Primary CatBoost model performance in temporal validation: discrimination (AUC) and decile calibration for 3-year **(A)** cancer-specific (AUC 0.701; calibration slope 1.12) and **(B)** overall (AUC 0.704; slope 1.08) mortality.

**Figure 3:**
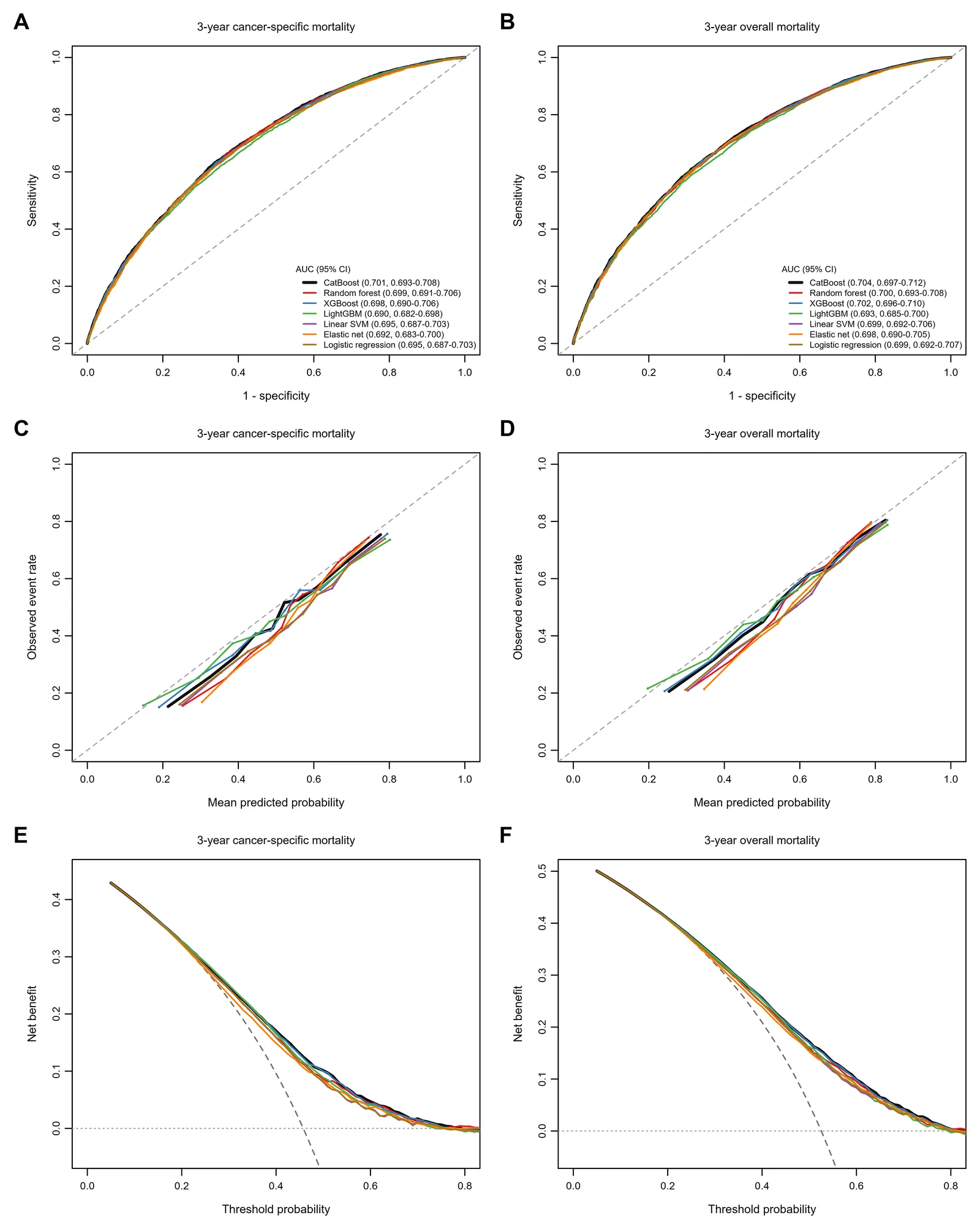
Multimodel comparison and clinical utility in temporal validation for the seven algorithms, for 3-year cancer-specific (left column) and overall (right column) mortality. **(A, B)** Receiver operating characteristic curves with areas under the curve and 95% confidence intervals; **(C, D)** decile calibration of predicted versus observed risk (dashed line, perfect calibration); **(E, F)** decision-curve net benefit (dashed gray line, treat all; dotted line, treat none). CatBoost, the primary model, is drawn in bold.

The original primary-model object was not retained; all results reported here derive from models retrained under the frozen specification, whose predictions correlate almost perfectly with the original development predictions (Spearman *r* ≈ 0.997) and which are the objects deposited for reuse. Throughout, the primary cancer-specific endpoint is a complete-case net probability of cancer death—a cause-specific quantity that excludes other-cause deaths—and not the absolute competing-risks cumulative incidence (**Supplementary Table 18**), whereas the OM endpoint is the absolute, all-cause quantity. In temporal validation, the diagnostic-time CatBoost model discriminated 3-year mortality with an AUC of 0.701 (95% CI 0.693–0.708) for CSM and 0.704 (0.697–0.712) for OM. Overall performance was sound (CSM: average precision 0.647, Brier 0.220; OM: average precision 0.707, Brier 0.219) and calibration was good (calibration slopes 1.12 and 1.08), with calibration plots showing close agreement between predicted and observed risk across the range (**Figure 2**). Extended calibration confirmed only mild over-prediction (observed-to-expected ratio 0.91 for CSM and 0.94 for OM; calibration-in-the-large intercept −0.19 and −0.15), with close predicted-versus-observed agreement across deciles of predicted risk (**Supplementary Table 11**; **Supplementary Figure 2**). Decision-curve analysis demonstrated positive net benefit across the threshold range examined, with the margin over flag-all widening across roughly 30%–70%—the range in which flag-all becomes uninformative or harmful and the model’s advantage is largest (**Figure 3**; **Supplementary Table 21**). Net benefit here is computed for the decision to flag a patient for intensified prognostic counseling, closer follow-up and trial referral—not for initiating or withholding systemic therapy—and the threshold probability is the 3-year mortality risk at which flagging is judged worthwhile. Because elderly men with metastatic disease also face substantial competing, non-cancer mortality, we emphasize that the primary cancer-specific endpoint is a complete-case net probability of cancer death, not the absolute competing-risks cumulative incidence; this competing-risks perspective is examined directly by the Fine–Gray and inverse-probability-of-censoring-weighted analyses (Sections 3.7 and 3.8; **Supplementary Tables 5**, **10**), in which cancer-specific cumulative/dynamic discrimination is expectedly lower (AUC 0.658 by IPCW and 0.676 by Fine–Gray), so the cancer-specific model is best read as a net-risk stratification tool.

### 3.3 Model comparison and the value proposition

Across the seven algorithms, discrimination was uniformly moderate in temporal validation (CSM AUC 0.690–0.701; OM AUC 0.693–0.704; **Table 2**; **Supplementary Table 1**), and CatBoost had the numerically highest AUC for both endpoints. In 1,000× paired bootstrap comparisons (**Supplementary Table 2**), the between-algorithm AUC differences were uniformly small (ΔAUC 0.002–0.011) and, although each reached statistical significance given the large validation sample, all were clinically negligible; CatBoost’s numerical advantage over the next-best algorithms—random forest and XGBoost—was only ΔAUC ≈ 0.002. CatBoost and XGBoost were the best-calibrated of the tree ensembles (calibration slope 1.12 and 1.02 for CSM; 1.08 and 1.03 for OM), whereas random forest over-shrank (1.34 and 1.30) and LightGBM over-extended (0.88 and 0.89). CatBoost and XGBoost were therefore effectively tied on the prespecified calibration and discrimination criteria, and CatBoost was selected on the final tier of the hierarchy—exact full-cohort SHAP interpretation and reproducibility—rather than on any material performance advantage. Because a conventional logistic model achieved closely similar discrimination (CSM 0.695, OM 0.699), the added value of the selected model lies not in higher discrimination but in its calibration, decision-curve utility, transparent interpretation, reproducibility, and retention of missing/unknown values as explicit categories. Against a parsimonious three-variable rule using only age, PSA category, and Gleason category—the information a clinician can apply without software—the primary model discriminated better for both endpoints (CSM 0.701 versus 0.671; OM 0.704 versus 0.680; DeLong *p* < 0.001), with higher decision-curve net benefit across the 30%–70% range, although the increment was modest (**Supplementary Table 27**). Multimodel receiver operating characteristic (ROC), calibration, and decision curves are shown in **Figure 3**.

**Table 2:** Discrimination and calibration of seven algorithms in temporal validation.

Model	CSM AUC	CSM AP	CSM Brier	CSM slope	OM AUC	OM AP	OM Brier	OM slope
Logistic regression	0.695	0.639	0.226	1.09	0.699	0.701	0.224	1.11
Elastic net	0.692	0.634	0.228	1.38	0.698	0.701	0.226	1.34
Random forest	0.699	0.643	0.224	1.34	0.700	0.702	0.222	1.30
XGBoost	0.698	0.645	0.220	1.02	0.702	0.705	0.219	1.03
LightGBM	0.690	0.637	0.223	0.88	0.693	0.696	0.222	0.89
CatBoost (primary)	**0.701**	**0.647**	**0.220**	**1.12**	**0.704**	**0.707**	**0.219**	**1.08**
Linear SVM (Platt)	0.695	0.639	0.226	1.12	0.699	0.701	0.224	1.16

Validation n = 16,806 (CSM)/19,122 (OM). AP, average precision; slope, calibration slope. Bold values are those of CatBoost, the primary model.

### 3.4 Model interpretation

**Figure 4:**
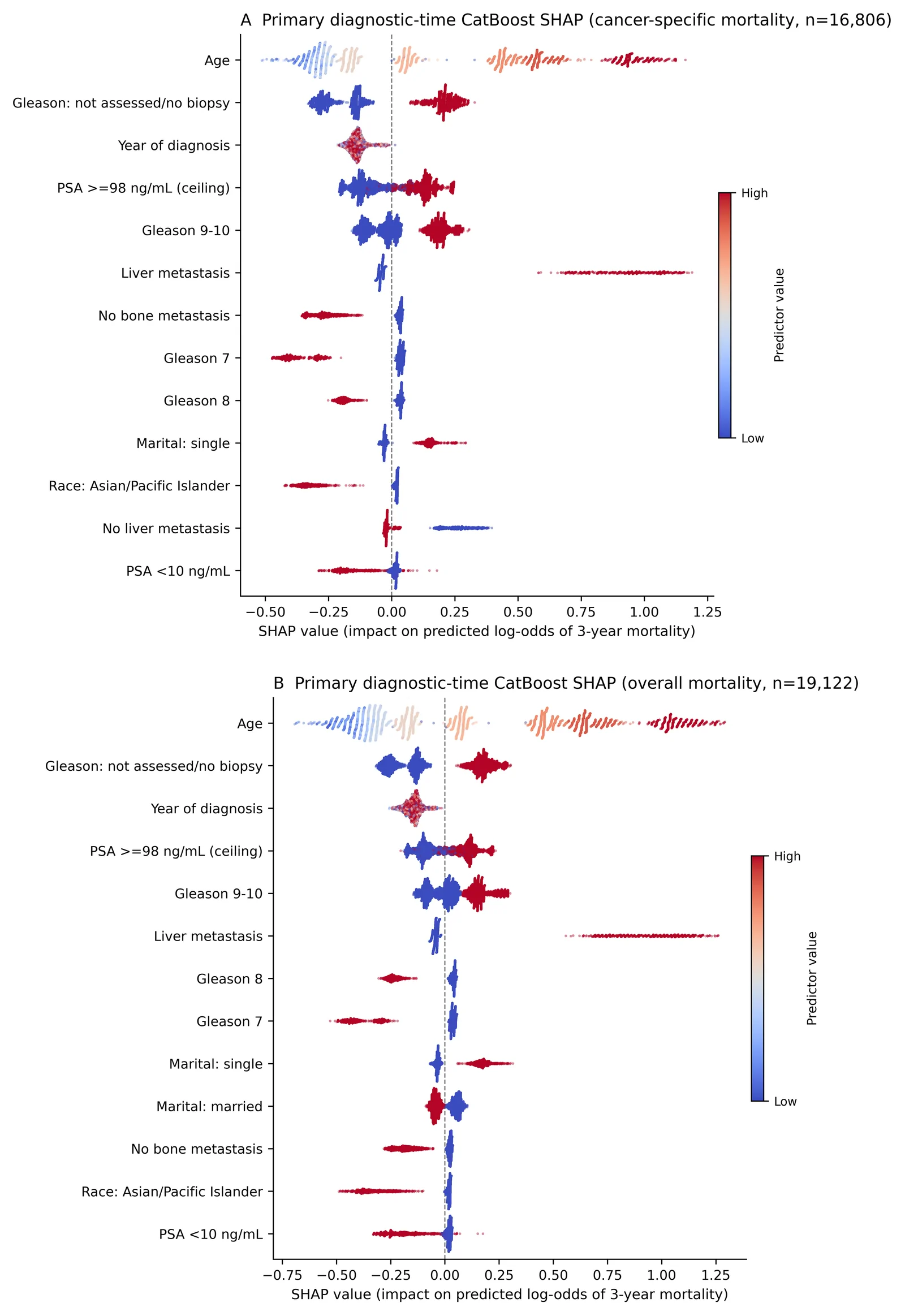
SHAP interpretation of the primary diagnostic-time CatBoost model over the entire validation cohort (cancer-specific *n* = 16,806; overall *n* = 19,122): SHAP beeswarm summary for **(A)** cancer-specific mortality and **(B)** overall mortality. Each point is a patient; predictors are ordered top-to-bottom by mean absolute SHAP value, the horizontal position is the SHAP value (impact on the predicted log-odds of 3-year mortality), and the color encodes the predictor’s value (blue, low; red, high), so both the magnitude and the direction of each effect are shown. Dominant contributors were age, the Gleason “not assessed/no biopsy” category, year of diagnosis, PSA, Gleason 9–10, and liver metastasis; older age, higher Gleason, higher PSA, and visceral (particularly liver) metastases increased predicted mortality, whereas more recent diagnosis year decreased it. Predictors are shown as one-hot-encoded categories (so, for example, a “No liver metastasis” row is a category rather than a separate variable); SHAP importance aggregated to the original variables is provided in **Supplementary Table 20**. Treatment variables were not included in the primary model.

Full validation-set SHAP analysis of the primary diagnostic-time model (no sampling; additivity verified) attributed predictions predominantly to age, Gleason score, year of diagnosis, PSA, and liver metastasis (**Figure 4**). For CSM, the five strongest contributors by mean |SHAP| were age, the “Gleason not assessed/no biopsy” category, year of diagnosis, PSA ≥98 ng/mL, and Gleason 9–10; the ranking was essentially identical for OM. Directionally, older age, higher Gleason, higher PSA, and visceral (particularly liver) metastases increased predicted mortality, whereas more recent diagnosis year was associated with lower predicted mortality. Notably, the “Gleason not assessed” category was itself among the strongest contributors, indicating that absent biopsy information is prognostically meaningful—plausibly marking patients too advanced or frail to undergo biopsy, or with an incomplete diagnostic workup, rather than representing ordinary random missingness—and supporting the decision to retain unknown values as explicit categories. Consistent with this, patients with unassessed Gleason were older, had higher PSA and more visceral metastases, were far less likely to undergo surgery, and had substantially higher 3-year mortality than those with assessed grade (**Supplementary Table 17**); because the meaning of an absent biopsy reflects local diagnostic and referral practice, the calibration of any model that uses this category should be re-established before use in other settings. The decline in predicted mortality with more recent diagnosis year is clinically plausible—consistent with the diffusion of treatment intensification and improved supportive care, and with documented real-world survival gains in *de novo* metastatic disease across 2010–2020 (34)—but, as a calendar-time variable, it may also partly reflect evolving SEER coding, stage migration, and the shorter maximum follow-up of recently diagnosed patients, and is therefore best read as a temporal signal rather than a modifiable patient factor. Overall, the SHAP profile recovers established prognostic biology (age, Gleason grade, PSA, and visceral—particularly liver—spread) without the model being given any treatment information. Part of the discrimination is nevertheless carried by a healthcare-process category (“Gleason not assessed/no biopsy”), a workup and frailty marker whose meaning—and therefore the model’s calibration—is local. Restricted to patients with an assessed Gleason score, in whom that missingness marker carries no information, discrimination was 0.685 (cancer-specific; *n* = 9,843, 3,576 events) and 0.682 (overall; *n* = 10,851, 4,608 events), only approximately 0.02 below the whole-cohort values, with better calibration than the unassessed stratum (slopes 1.08 and 1.02 versus 0.91 and 0.91; **Supplementary Table 12**), indicating that substantial genuine tumor-biology signal persists once the healthcare-process marker is removed.

### 3.5 Risk stratification

**Figure 5:**
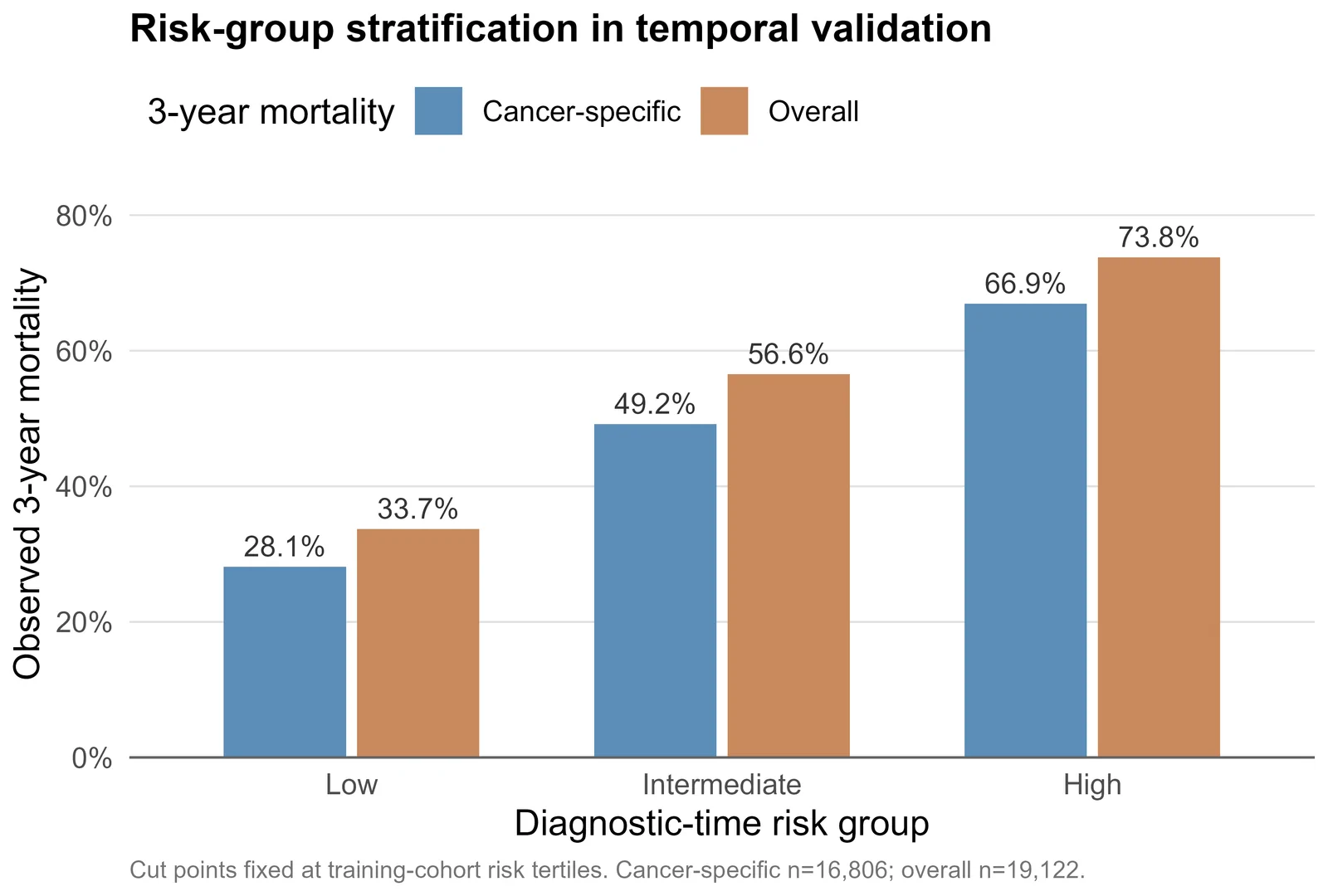
Risk-group stratification in temporal validation: observed 3-year cancer-specific and overall mortality by diagnostic-time risk group (cut points fixed at training-cohort risk tertiles).

Cut points fixed at training-cohort risk tertiles separated the validation cohort into groups with monotonically increasing observed mortality: for CSM, 28.1%/49.2%/66.9% (low/intermediate/high), and for OM, 33.7%/56.6%/73.8% (**Figure 5**). Expressed as absolute competing-risks quantities, the 36-month Aalen–Johansen cumulative incidence of cancer death across the cancer-specific risk strata—computed on all 19,473 validation patients—was 25.7%, 42.9%, and 54.7% (a 29-percentage-point low-to-high gap), while competing other-cause mortality rose from 7.9% to 18.0% (**Supplementary Table 26**). The separation is large in absolute terms—an approximately 39–40-percentage-point gap in observed 3-year mortality between the low- and high-risk groups for both overall (33.7% vs. 73.8%) and cancer-specific (28.1% vs. 66.9%) death—so that, despite only moderate discrimination, the model resolves clinically distinct prognostic strata that are potentially useful within the SEER-like setting for counseling and for enriching trials with high-risk patients. This is a clear risk gradient in temporal SEER validation.

### 3.6 Secondary treatment-associated analyses (observational)

Adding SEER-documented treatment variables produced negligible incremental discrimination over the diagnostic-time model (CatBoost ΔAUC ≤0.002; logistic ΔAUC ≤0.001; **Supplementary Table 3**), supporting their exclusion from the diagnostic-time model; exploratory within-risk-group treatment associations are confined to the **Supplementary Material** (**Supplementary Figure 1**; **Supplementary Tables 4**, **7**). Because SEER does not capture androgen-deprivation therapy or androgen-receptor pathway inhibitors and records chemotherapy only as documented “yes” versus “no/unknown”, these analyses are not interpreted as treatment effects.

### 3.7 Survival-modeling sensitivity analyses

Time-to-event models on the same diagnostic-time predictors gave broadly consistent moderate discrimination and did not outperform the primary fixed-time model: Cox OM C-index 0.656 (3-year AUC 0.698); cause-specific Cox CSM C-index 0.653 (3-year AUC 0.694); Fine–Gray competing-risk CSM C-index 0.639 (3-year AUC 0.676, Brier 0.230); random survival forest C-index 0.650 (OM) and 0.647 (CSM).

### 3.8 Robustness

The main conclusions were robust. Retaining 0-month survival cases slightly increased discrimination (CSM AUC 0.701→0.711; OM 0.704→0.715; **Supplementary Table 6**). Performance was broadly consistent across prespecified subgroups—age, race, PSA and Gleason known/unknown, visceral versus non-visceral metastasis, and diagnosis year (**Supplementary Table 12**)—with lower discrimination where prognostic information was limited: unknown Gleason (CSM AUC 0.633; OM 0.642), the oldest patients (age ≥80 years; CSM 0.648; OM 0.645), and the small American Indian/Alaska Native subgroup; calibration was stable across most strata. Handling unknown PSA and Gleason as explicit categories outperformed both complete-case analysis (CSM AUC 0.701→0.685; OM 0.704→0.682) and simple mode imputation (CSM 0.697; OM 0.700), supporting retention of unknown categories (**Supplementary Table 13**). Formal multiple imputation under missing-at-random and a pattern-mixture (missing-not-at-random) tipping-point analysis were concordant, reproducing the complete-case discrimination and preserving the risk-group gradient across plausible missing-not-at-random departures (**Supplementary Table 15**). Inverse-probability-of-censoring-weighted fixed-time validation on the entire validation cohort indicated that early censoring did not materially distort the OM estimand, which was concordant with the complete-case analysis (IPCW AUC 0.703; Brier 0.219 at 36 months). For CSM, the IPCW analysis targets a different (absolute competing-risks) estimand than the primary complete-case net-risk model; its lower cause-specific cumulative/dynamic AUC (0.658), with predicted net risk exceeding the competing-risks cumulative incidence, is expected for absolute competing-risk prediction and directionally supports the model as a net-risk stratification tool rather than confirming robustness to competing mortality (**Supplementary Table 10**). Landmark (3- and 6-month) and strict treatment-coding analyses were concordant with the primary results (**Supplementary Table 6**). Removing year of diagnosis left discrimination and average precision essentially unchanged (cancer-specific AUC 0.701 versus 0.701 and average precision 0.647 versus 0.647; overall 0.704 versus 0.704 and 0.707 versus 0.708) but produced a small, consistent loss of overall fit and calibration (Brier 0.220 to 0.224 and 0.219 to 0.222; calibration slope 1.115 to 1.130 and 1.079 to 1.095) and shifted absolute risk-group rates downward (cancer-specific 28%/49%/67% to 25%/46%/64%; overall 34%/57%/74% to 31%/53%/72%), consistent with year of diagnosis carrying calendar-time information about declining baseline risk rather than adding rank-ordering signal (**Supplementary Table 25**). The rank ordering is therefore preserved without year of diagnosis while the absolute level shifts, which is precisely what local intercept-and-slope recalibration corrects; a year-free model would require re-derived risk-group cut points and recalibration before use.

### 3.9 Temporal transportability (internal–external cross-validation)

In leave-one-calendar-year-out internal–external cross-validation across the 11 diagnosis years (each held-out year contributing at least 1,200 CSM and 1,466 OM events), pooled discrimination matched the primary model: pooled AUC was 0.701 for CSM and 0.703 for OM (year-cluster bootstrap 95% CI 0.693–0.708 and 0.694–0.710; the naive patient-level interval is anti-conservative because folds share training data, see **Supplementary Table 22**), with calibration slopes 1.02 and 1.04 and calibration-in-the-large near zero (**Supplementary Table 22**). A DerSimonian–Laird random-effects meta-analysis gave a summary AUC of 0.697 (95% prediction interval 0.672–0.721; *I*² 54%) for CSM and 0.699 (95% prediction interval 0.673–0.725; *I*² 61%) for OM; per-year AUC ranged from 0.670 to 0.716 (cancer-specific) and 0.675 to 0.720 (overall), and the prediction interval excluded any non-discriminating year (**Supplementary Figure 3**). In the secondary prospective rolling-origin analysis, which trained only on prior years, pooled AUC was 0.696 (cancer-specific) and 0.699 (overall) with calibration slopes 0.97 and 1.00; performance was weakest in the data-poor earliest years and strengthened from 2015 onward (2019–2020 AUC 0.711–0.716 cancer-specific and 0.715–0.720 overall). Absolute 3-year mortality declined across the decade (cancer-specific 56% to 45%; overall 61% to 53%), yet per-year calibration-in-the-large remained near zero, indicating preserved calibration despite falling baseline risk. Because year of diagnosis is retained as a model feature, the leave-one-year scheme interpolates the held-out year, and this potential optimism is bounded by the rolling-origin result; this is within-SEER temporal transportability and does not establish geographic generalizability across registries.

### 3.10 External validation in an independent, non-SEER cohort

Applied without any modification to a geographically and ethnically distinct single-center Chinese cohort that shares no patients, registry, or coding system with SEER (129 CSM-evaluable and 146 OM-evaluable men; observed 3-year mortality 50% and 56%), the locked models showed attenuated discrimination relative to internal validation (cancer-specific AUC 0.59, 95% CI 0.49–0.69; overall AUC 0.59, 95% CI 0.50–0.68); because the lower confidence bounds reached approximately 0.5, we make no claim of statistically significant external discrimination. The prespecified, frozen risk-group cut points nonetheless produced limited separation of observed risk that was clearest for OM (low 51%, intermediate 65%, high 71%, a monotone gradient); for CSM, the low-risk group (44%) remained well below the higher groups, but the intermediate and high groups did not themselves separate (64% and 63%; only 25 and 16 patients), so the full risk gradient transported clearly only for OM (**Supplementary Table 23**). Calibration drifted in the expected direction on transport: predictions were too extreme (calibration slope 0.47 for both endpoints) and modestly under-estimated absolute risk in this higher-mortality cohort (O:E 1.23 and 1.19; calibration-in-the-large intercept +0.42 and +0.40; **Supplementary Figure 4**), so local intercept-and-slope recalibration would be required before any use outside the development setting. This attenuation is plausibly explained, at least in part, by the markedly different case mix of a single-center referral cohort (**Supplementary Table 24**): although the burden of bone metastases was similar (87%), the external cohort had a narrower PSA distribution and far less missing Gleason information (not assessed in 22.7% versus 43.2% of the SEER cohort), so the model could draw on less of its most discriminating diagnostic-time signal—including the prognostically important “Gleason not assessed” marker. The direction of this difference is instructive rather than simply explanatory: within SEER, discrimination was in fact higher in the assessed-Gleason stratum (0.685 and 0.682; **Supplementary Table 12**), so more complete grading should not, by itself, lower discrimination. What changes is the composition of the signal—the model’s learned use of the “Gleason not assessed” marker is calibrated to US registry workup practice and has little purchase here, while the remaining tumor-biology predictors are compressed into a narrower range (PSA concentrated in the 20–97.9 ng/mL band; 46% Gleason 9–10). This spectrum and case-mix compression, alongside the population, treatment-era, and information-ceiling differences discussed below, is the more plausible account of the attenuation. A further, structural assumption is that every patient in this mainland-Chinese cohort had to be mapped onto the model’s only Asian race code, the US SEER “Asian or Pacific Islander” category—a heterogeneous US-defined grouping whose SEER-estimated prognostic contribution need not carry over to a Chinese population—so this coding mismatch is itself a plausible contributor to the observed attenuation and calibration drift. On a single-center sample of this size, however, a genuine loss of transportability cannot be excluded. These results are therefore best read as a preliminary out-of-SEER external check: the model’s risk ordering partly transports (clearly for OM), but discrimination is attenuated, calibration must be updated locally, and larger multicenter, multi-ethnic validation with recalibration remains necessary before clinical deployment.

## 4 Discussion

We developed and temporally validated interpretable ML models for fixed 3-year CSM and OM in a large national cohort of *de novo* mPCa. The primary diagnostic-time CatBoost model achieved moderate discrimination (AUC ≈ 0.70), acceptable calibration, and positive net benefit on decision-curve analysis; discrimination was stable across a 4-year temporal shift, and the model separated patients into risk groups with a steep mortality gradient. Full validation-set SHAP provided a transparent account of predictions dominated by age, Gleason score, PSA, and metastatic sites.

Discrimination of approximately 0.70 is consistent with, and not superior to, previously reported nomograms for mPCa (15, 16) and a conventional logistic model performed comparably in our own benchmark. We therefore do not claim that algorithmic complexity improves discrimination. A ceiling on discrimination is in fact expected here: SEER lacks the variables that most strongly stratify mPCa prognosis—alkaline phosphatase, lactate dehydrogenase, hemoglobin, performance status, and formal disease-volume (high- versus low-volume) criteria—as well as the androgen-deprivation and androgen-receptor pathway treatment data that shape outcome, so no model trained on these inputs could be expected to achieve high discrimination, and the comparable performance of a simple logistic model is consistent with an information ceiling rather than a modeling failure. The contribution is instead methodological: an explicitly interpretable, well-calibrated, decision-curve-positive diagnostic-time tool, developed and reported under TRIPOD+AI, with transparent retention of missing/unknown values as explicit categories. In a uniformly metastatic case mix that compresses the achievable predictor range, calibration and interpretability—rather than marginal gains in discrimination—are the properties that determine whether a risk tool is usable in practice (28).

These choices also distinguish the present work from many earlier prognostic models in mPCa. Prior SEER-based nomograms and single-algorithm ML studies have frequently relied on random rather than temporal data splits, incorporated post-diagnosis treatment variables into the predictor set, reported discrimination with limited attention to calibration or decision-curve utility, and supplied neither model interpretation nor runnable code (15, 16, 18, 35); **Supplementary Table 14** summarizes how these prior models compare with the present study on data source, validation design, calibration, decision-curve analysis, interpretability, and code availability. We instead use a temporally independent validation set, exclude treatment from the diagnostic-time model, report calibration and net benefit alongside discrimination, define the estimand explicitly, interpret the model with full validation-set SHAP, and release the analysis code—prioritizing transparent, transportable prognostication over headline accuracy.

Adding SEER-documented treatment variables yielded negligible incremental discrimination, and treatment contributions were small in SHAP. This null result must be read in light of a fundamental data limitation: SEER does not record androgen-deprivation therapy or androgen-receptor pathway inhibitors, which—frequently combined with docetaxel—constitute the backbone of contemporary metastatic hormone-sensitive prostate cancer therapy (3–8, 10), as reflected in current treatment guidelines (12, 13), and even chemotherapy is captured only as documented “yes” versus “no/unknown”. The treatment information visible to SEER is therefore a small and incomplete fraction of the systemic therapy that actually shapes outcome, and the near-null increment is expected rather than surprising. Real-world analyses confirm that treatment intensification has risen over this period, alongside gradually improving survival in *de novo* metastatic disease (34, 36); none of that intensification is visible in the SEER treatment fields, which record neither androgen-deprivation therapy nor androgen-receptor pathway inhibitors. The risk-stratified treatment associations we report (for example, chemotherapy-by-risk heterogeneity) are exploratory and observational, vulnerable to confounding by indication and to the no/unknown coding, and must not be interpreted as treatment effects; we therefore report them descriptively, draw no causal or comparative-effectiveness conclusions, and present them only as hypotheses to be tested in studies with complete systemic-therapy data.

We therefore position the model as a diagnostic-time tool for risk stratification and communication rather than for treatment selection, for which it is neither designed nor validated. Its intended uses are concrete: at the time of diagnosis, it translates an individual patient’s profile into an individualized, calibrated 3-year OM estimate and net cancer-specific risk stratification to support honest prognostic counseling with patients and families, to inform the intensity of follow-up and surveillance, and to enrich or stratify clinical trials. It is equally important to state what the model is not for: it must not be used to select or withhold systemic therapy, to override clinical judgment, or—without prior local recalibration—to generate absolute risks for populations outside the SEER development setting. This caution applies with particular force to the missing-indicator variables: because a large fraction of patients had no assessed Gleason score and this category is both strongly prognostic and a marker of local diagnostic and referral practice (**Supplementary Table 17**), its meaning, and therefore the model’s calibration, is unlikely to transport across hospitals or countries without local recalibration. Because predictive performance—particularly calibration—can degrade when a model is applied to a population different from the one in which it was developed, any application beyond the development setting should be preceded, at minimum, by local intercept-and-slope recalibration, with calibration and decision-curve net benefit re-assessed in the target population before any deployment. Because year of diagnosis is a calendar-time variable, the model should not be applied to patients diagnosed after the 2010–2020 development window without local recalibration; a sensitivity analysis omitting year of diagnosis confirmed that the rank ordering is preserved without it, while the absolute risk level shifts (**Supplementary Table 25**), so a year-of-diagnosis-free model would require re-derived risk-group cut points and local recalibration before use rather than being a drop-in substitute. Consistent with this use, decision-curve analysis showed positive net benefit across roughly 30%–70% threshold probabilities; in a disease with this high baseline 3-year mortality, that range plausibly spans the risk thresholds at which prognostic counseling, follow-up intensity, and trial-enrichment decisions are actually made, so the net-benefit result reflects potential clinical utility rather than a purely methodological flourish. The steep, reproducible risk gradient is well suited to identifying a high-risk stratum for trials of treatment intensification.

Strengths include a large national cohort; a temporal rather than random-split validation that tests performance on later, unseen patients; benchmarking of seven algorithms under a unified pipeline; full validation-set SHAP computed without sampling or approximation; survival-model sensitivity analyses (Cox and random survival forest) concordant with the primary fixed-time model and an explicit competing-risks (Fine–Gray and IPCW) analysis of the distinct absolute-incidence estimand; an extensive prespecified robustness battery; appraisal against PROBAST+AI for risk of bias and applicability (20); and disciplined avoidance of overclaiming, including no causal treatment inference and no claim of algorithmic superiority. The independent external validation reported here is presented as a preliminary, single-center check rather than as proof of transportable performance.

This study also has important limitations. It is retrospective and registry-based. Beyond temporal validation, we performed an initial independent external validation in a geographically and ethnically distinct, non-SEER single-center Chinese cohort (Section 3.10): the risk-group ordering partly transported—most clearly for OM—but discrimination was attenuated (AUC ≈ 0.59, CIs approaching 0.5) and calibration drifted (slope 0.47; under-prediction of absolute risk), so the model requires local intercept-and-slope recalibration and remains to be validated in larger, multicenter and multi-ethnic cohorts before non-SEER use; the wide single-center CIs limit firm inference, and mapping a mainland-Chinese cohort onto the US SEER “Asian or Pacific Islander” race category is itself a transportability assumption that may contribute to the attenuation. To probe transportability across the diagnosis-year axis, we added a fully data-complete temporal internal–external cross-validation (leave-one-calendar-year-out across 2010–2020, with a prospective rolling-origin complement); discrimination and calibration were stable across all 11 diagnosis years (pooled AUC ≈ 0.70; random-effects 95% prediction interval 0.672–0.721 for CSM and 0.673–0.725 for OM, excluding any non-discriminating year), with between-year heterogeneity that was moderate in relative terms (*I*² 54%–61%) but small in absolute terms. This assesses transportability across diagnosis periods within SEER and does not establish geographic or cross-system generalizability, which remains untested because every fold shares the same registries, coding manual, and US case mix. A registry-level geographic internal–external cross-validation was not feasible because the SEER registry identifier is suppressed in the SEER Research and Research Limited-Field products available to us and is released only through SEER Research Plus, to which we had no access; the deposited leave-one-registry-out code is runnable should registry-level data become available. SEER lacks established prognostic variables such as alkaline phosphatase, lactate dehydrogenase, hemoglobin, performance status, and formal disease-volume criteria, which both limits achievable discrimination and constrains transportability. PSA is subject to SEER recode conventions: because ≥98 ng/mL is a top-coded ceiling category, PSA is effectively dichotomized for 46.8% of the cohort, truncating the upper end of one of the model’s main predictors and compressing achievable discrimination, and the SEER 2010+ PSA lab-value recode is further affected by year-to-year coding changes, so PSA here is best read as a coarse ordinal marker rather than a precise laboratory value. Gleason was unassessable in a substantial minority (41.6%). This missingness is very unlikely to be random: an absent biopsy or PSA most plausibly marks patients who are more advanced, frailer, or incompletely worked up, so the “Gleason not assessed” category behaves as an informative prognostic marker (Section 3.4) rather than a neutral gap, carrying a potential selection and ascertainment bias that no imputation can fully remove. We therefore used a missing-indicator approach (retaining unknown values as explicit categories), which is appropriate when missingness is itself informative, and bracketed its impact with complete-case and simple-imputation sensitivity analyses (**Supplementary Table 13**). Multiple imputation under a missing-at-random assumption reproduced the complete-case discrimination (pooled validation AUC, approximately 0.68) and stayed below the missing-indicator primary, and a pattern-mixture (missing-not-at-random) tipping-point analysis that progressively shifted the unknown-Gleason patients toward high grade did not degrade discrimination or the risk-group gradient (**Supplementary Table 15**), indicating that the findings are robust to plausible departures from missing-at-random. CSM depends on registry cause-of-death coding and may be misclassified. Histology was not restricted during cohort selection, so the cohort comprises malignant prostate tumors of all types; although these are overwhelmingly adenocarcinoma, rare aggressive variants such as small-cell or neuroendocrine carcinoma have distinct biology and prognosis. On a re-extraction that added histology, adenocarcinoma comprised 85.6% of the cohort, carcinoma or malignant neoplasm not otherwise specified made up 13.0%, and specified non-adenocarcinoma variants comprised only 1.5% (small-cell or neuroendocrine carcinoma 1.2%); an adenocarcinoma-restricted sensitivity analysis reproduced the primary findings with modestly lower discrimination (validation AUC, 0.687 cancer-specific and 0.688 overall) and maintained calibration (**Supplementary Table 16**), indicating the results are not driven by rare aggressive variants. The primary cancer-specific endpoint is a complete-case, cause-specific net probability of cancer death and should be interpreted as a net-risk stratification target, not as the absolute cumulative incidence of cancer death in the presence of competing mortality; the inverse-probability-of-censoring-weighted competing-risks analysis (**Supplementary Table 10**) addresses that distinct and intrinsically harder target and, as expected, showed lower discrimination, reinforcing that the primary model is a net-risk tool rather than an absolute competing-risk predictor. Put plainly, a high cancer-specific score flags a patient at high relative risk of prostate-cancer death for counseling and triage; it does not represent that patient’s absolute probability of dying from prostate cancer once competing causes of death are taken into account (**Supplementary Table 18**). The original primary-model object was not retained and was reconstructed from the frozen specification, closely reproducing the original predictions; to support exact reuse, the retrained model objects are deposited together with their seed, hyperparameters, feature list, risk-group cut points, and a SHA-256 model checksum. The survival-model results are reconstructed sensitivity analyses and should not be over-interpreted. Finally, the treatment analyses are observational and constrained by the SEER treatment fields as described, and SEER identifies *de novo* metastatic disease but does not record castration-sensitive status, androgen-deprivation therapy, or androgen-receptor pathway inhibitor exposure; the cohort is therefore defined by *de novo* metastatic presentation, and “hormone-sensitive” is used only as the conventional clinical descriptor of this predominantly treatment-naive-at-diagnosis population rather than an individually verified state.

## 5 Conclusion

In a large national cohort of *de novo* mPCa, an interpretable diagnostic-time ML model provided temporally validated, well-calibrated 3-year OM risk and net CSM-risk stratification, with a clear risk-group gradient and positive clinical net benefit for flagging higher-risk patients. These findings hold within the 2010–2020 SEER development window: because year of diagnosis is a model predictor and baseline mortality fell across the decade, the model should not be applied to patients diagnosed after that window without local recalibration. The cancer-specific estimate is a net, cause-specific risk for stratification, not the absolute probability of dying from prostate cancer once competing causes of death are taken into account (**Supplementary Table 18**); the OM estimate is the absolute, bedside-interpretable number.

Transportability beyond SEER was, on present evidence, limited. Applied without modification to an independent non-SEER single-center cohort, the locked model transported only partially: discrimination fell to an AUC of 0.59 (95% CI 0.49–0.69 cancer-specific and 0.50–0.68 overall), the calibration slope fell to 0.47 with under-prediction of absolute risk (O:E 1.23 and 1.19), and only the OM risk ordering was preserved as a monotone gradient. The model should therefore not be applied outside the SEER development setting without local intercept-and-slope recalibration, and on a single-center sample of this size, a genuine loss of transportability cannot be excluded.

Discrimination was comparable to that of simpler models and existing nomograms, so the model’s value lies in calibrated, transparent, diagnostic-time risk stratification rather than in superior discrimination or treatment guidance; the aim is not to show that ML outperforms conventional models but to provide a transparent, calibrated, interpretable, and reproducible diagnostic-time risk tool. SEER-documented treatment added little and cannot represent contemporary systemic therapy, so treatment-associated findings are hypothesis-generating only. Within the limits set out above, the model is best used for risk communication, calibration of follow-up intensity, and trial enrichment, not for treatment selection; the locked model objects, their feature-order snapshot, and the frozen risk-group cut points are openly deposited under an MIT license, so that other groups can score their own patients subject to the local-recalibration requirement.

Building on this initial single-center external validation, the priority next step is larger, multicenter, and multi-ethnic external validation with local recalibration in real-world cohorts that capture the prognostic variables SEER lacks—performance status [Eastern Cooperative Oncology Group (ECOG)], alkaline phosphatase, lactate dehydrogenase, hemoglobin, and formal disease-volume criteria—together with detailed systemic therapy (androgen-deprivation therapy, androgen-receptor pathway inhibitors, and docetaxel); subsequent work could then assess whether the model can be deployed as an online calculator or an electronic-health-record risk alert to support point-of-care use, a step that would itself require prospective, early-stage clinical evaluation (37).

## Data Availability

Publicly available datasets were analyzed in this study. This data can be found here: Surveillance, Epidemiology, and End Results (SEER) Program, U.S. National Cancer Institute: https://seer.cancer.gov/data/-. The SEER Research Data are available to investigators who sign the SEER Research Data Use Agreement, and the authors are not permitted to redistribute the individual-level data. The individual-level records of the independent external-validation cohort (The Second People’s Hospital of Wuhu) are not publicly available owing to patient-privacy and institutional data-governance restrictions but are available from the corresponding author on reasonable request. The analysis code, run scripts, predictor dictionary, and the retrained primary model objects with their reproducibility snapshots that reproduce the tables and figures are openly available in a public Zenodo repository: https://doi.org/10.5281/zenodo.21583580 (v1.9.0).

## Supplementary material

Supplementary Figure 1 Risk-stratified treatment-associated mortality differences (observational): stabilized-IPTW weighted risk differences (treated minus comparator) by treatment and risk group for cancer-specific (A) and overall (B) mortality. Markers denote cautious (open triangle) or weak/unstable (asterisk) signals. Observational association only; comparators are documented treatment versus no/unknown.

Supplementary Figure 2 Decile calibration of the primary diagnostic-time CatBoost model (predicted vs observed 3-year risk).

Supplementary Figure 3 Temporal internal–external cross-validation forest plot: per-year discrimination with random-effects pooling.

Supplementary Figure 4 External-cohort calibration of the primary models (predicted vs observed 3-year cancer-specific and overall mortality in the independent, non-SEER single-center cohort).
