# Supplementary material for: Interpretable machine-learning risk stratification at the time of diagnosis for 3-year mortality in de novo metastatic prostate cancer: development and temporal validation in SEER
Source: Front Oncol. 2026 Aug 31;16:1904589. doi: 10.3389/fonc.2026.1904589 (PMC13572273; doi:10.3389/fonc.2026.1904589)
Supplement: Supplementary file 1 [file DataSheet1.docx]

# Supplementary Material

Temporal validation cohort: cancer-specific (CSM) n=16,806; overall (OM) n=19,122.

## Supplementary Methods. Cohort definition and SEER*Stat case selection

Data source: SEER Research Data, 17 registries, November 2025 submission (released April 2026; U.S. National Cancer Institute). The cohort comprised men with malignant prostate cancer (primary site C61.9) and distant disease at diagnosis—defined by the Combined Summary Stage with Expanded Regional Codes (2004+) value “Distant site(s)/node(s) involved”—diagnosed 2010–2020, with the SEER*Stat “Select Only” filters for malignant behavior and known age. Histology was not further restricted, so the cohort comprises malignant prostate tumours of all histologies (overwhelmingly adenocarcinoma). The analytic export retained the variables listed in Table S9; the exact SEER*Stat case-selection statement is reproduced verbatim below for transparency and independent audit.

SEER*Stat “Select Only” filters: **Malignant Behavior** and **Known Age**. Selection statement (verbatim):

{Race, Sex, Year Dx.Year of diagnosis} = '2010','2011','2012','2013','2014','2015','2016','2017','2018','2019','2020'
AND {Site and Morphology.Primary Site - labeled} = 'C61.9-Prostate gland'
AND {Stage - Summary/Historic.Combined Summary Stage with Expanded Regional Codes (2004+)} = 'Distant site(s)/node(s) involved'

Analytic exclusions applied to the export: cases with survival shorter than one month (1,606 with <1 month and 28 with missing survival time) were excluded to avoid unstable or ambiguous diagnosis-month records, yielding the main analytic cohort of 41,057; retention of 0-month cases was assessed as a sensitivity analysis. The SEER registry identifier is suppressed in the SEER Research Data and Research Limited-Field products and is released only through SEER Research Plus, to which the authors did not have access, so registry-level (geographic) internal–external validation could not be performed; a temporal internal–external cross-validation was performed instead (Section 2.10, Table S22).

## Table S1. Per-model discrimination and overall performance in temporal validation

| Endpoint | Model | AUC (95% CI) | Average precision | Brier |
| --- | --- | --- | --- | --- |
| CSM | CatBoost (primary) | 0.701 (0.693–0.708) | 0.647 | 0.220 |
| CSM | Random forest | 0.699 (0.691–0.706) | 0.643 | 0.224 |
| CSM | XGBoost | 0.698 (0.691–0.706) | 0.645 | 0.220 |
| CSM | Linear SVM | 0.695 (0.687–0.703) | 0.639 | 0.226 |
| CSM | Logistic regression | 0.695 (0.687–0.703) | 0.639 | 0.226 |
| CSM | Elastic net | 0.692 (0.683–0.700) | 0.634 | 0.228 |
| CSM | LightGBM | 0.690 (0.683–0.698) | 0.637 | 0.223 |
| OM | CatBoost (primary) | 0.704 (0.697–0.712) | 0.707 | 0.219 |
| OM | XGBoost | 0.702 (0.696–0.710) | 0.705 | 0.219 |
| OM | Random forest | 0.700 (0.693–0.708) | 0.702 | 0.222 |
| OM | Logistic regression | 0.699 (0.692–0.707) | 0.701 | 0.224 |
| OM | Linear SVM | 0.699 (0.692–0.706) | 0.701 | 0.224 |
| OM | Elastic net | 0.698 (0.690–0.705) | 0.701 | 0.226 |
| OM | LightGBM | 0.693 (0.685–0.700) | 0.696 | 0.222 |

*AUC 95% CIs are from 1,000× stratified bootstrap; average precision and Brier are point estimates. Calibration slopes are in main-text Table 2.*

## Table S2. Pairwise bootstrap AUC differences, primary CatBoost versus each comparator

| Endpoint | Comparator | ΔAUC (CatBoost − comparator) | p |
| --- | --- | --- | --- |
| CSM | Random forest | 0.002 | 0.022 |
| CSM | XGBoost | 0.002 | <0.001 |
| CSM | Logistic regression | 0.006 | <0.001 |
| CSM | Linear SVM | 0.006 | <0.001 |
| CSM | Elastic net | 0.008 | <0.001 |
| CSM | LightGBM | 0.011 | <0.001 |
| OM | XGBoost | 0.002 | <0.001 |
| OM | Random forest | 0.004 | <0.001 |
| OM | Logistic regression | 0.005 | <0.001 |
| OM | Linear SVM | 0.005 | <0.001 |
| OM | Elastic net | 0.006 | <0.001 |
| OM | LightGBM | 0.011 | <0.001 |

*All pairwise differences favour CatBoost and are statistically significant given the large validation sample, but are clinically negligible in magnitude (ΔAUC ≤0.011).*

## Table S3. Incremental value of SEER-documented treatment variables (diagnostic-only vs treatment-augmented)

| Endpoint | Model | AUC diagnostic-only | AUC + treatment | ΔAUC |
| --- | --- | --- | --- | --- |
| CSM | Logistic regression | 0.695 | 0.695 | 0.000 |
| CSM | CatBoost | 0.701 | 0.702 | 0.001 |
| OM | Logistic regression | 0.699 | 0.700 | 0.001 |
| OM | CatBoost | 0.704 | 0.706 | 0.002 |

*Treatment variables add negligible discrimination; the largest treatment-variable contributor in SHAP of the treatment-augmented model was chemotherapy, which nonetheless remained secondary to diagnostic-time factors.*

## Table S4. Within-risk-group stabilized-IPTW treatment associations and balance/weight diagnostics (observational)

| Endpoint | Risk group | Treatment | Treated n | Comparator n | Weighted RD | Residual SMD (max) | Max stab. weight | Flag |
| --- | --- | --- | --- | --- | --- | --- | --- | --- |
| CSM | Low | Chemotherapy | 2,338 | 10,479 | +0.042 | 0.183 | 11.5 | residual SMD >0.1 |
| CSM | Low | Radiation | 3,521 | 9,296 | −0.028 | 0.109 | 3.1 | residual SMD >0.1 |
| CSM | Low | Surgery | 1,762 | 11,055 | +0.029 | 0.051 | 7.3 | — |
| CSM | Low | Any local therapy | 4,818 | 7,999 | −0.013 | 0.103 | 3.2 | residual SMD >0.1 |
| CSM | Intermediate | Chemotherapy | 2,334 | 9,875 | −0.051 | 0.225 | 8.9 | residual SMD >0.1 |
| CSM | Intermediate | Radiation | 3,137 | 9,072 | +0.003 | 0.048 | 5.2 | — |
| CSM | Intermediate | Surgery | 1,135 | 11,074 | +0.033 | 0.102 | 11.6 | residual SMD >0.1 |
| CSM | Intermediate | Any local therapy | 4,035 | 8,174 | +0.020 | 0.024 | 2.7 | — |
| CSM | High | Chemotherapy | 1,062 | 9,723 | −0.027 | 0.120 | 8.4 | residual SMD >0.1 |
| CSM | High | Radiation | 2,031 | 8,754 | +0.003 | 0.030 | 5.6 | — |
| CSM | High | Surgery | 886 | 9,899 | −0.054 | 0.129 | 10.7 | residual SMD >0.1 |
| CSM | High | Any local therapy | 2,769 | 8,016 | −0.011 | 0.041 | 4.8 | — |
| OM | Low | Chemotherapy | 2,676 | 11,708 | +0.021 | 0.214 | 9.5 | residual SMD >0.1 |
| OM | Low | Radiation | 3,945 | 10,439 | −0.036 | 0.115 | 3.8 | residual SMD >0.1 |
| OM | Low | Surgery | 1,911 | 12,473 | +0.035 | 0.044 | 7.6 | — |
| OM | Low | Any local therapy | 5,361 | 9,023 | −0.016 | 0.103 | 2.9 | residual SMD >0.1 |
| OM | Intermediate | Chemotherapy | 2,372 | 11,404 | −0.070 | 0.207 | 10.7 | residual SMD >0.1 |
| OM | Intermediate | Radiation | 3,383 | 10,393 | −0.016 | 0.035 | 4.7 | — |
| OM | Intermediate | Surgery | 1,236 | 12,540 | +0.014 | 0.098 | 13.0 | — |
| OM | Intermediate | Any local therapy | 4,367 | 9,409 | −0.004 | 0.019 | 2.8 | — |
| OM | High | Chemotherapy | 1,047 | 11,277 | −0.044 | 0.158 | 7.8 | residual SMD >0.1 |
| OM | High | Radiation | 2,180 | 10,144 | −0.005 | 0.029 | 5.7 | — |
| OM | High | Surgery | 1,069 | 11,255 | −0.037 | 0.122 | 8.2 | residual SMD >0.1 |
| OM | High | Any local therapy | 3,079 | 9,245 | −0.011 | 0.041 | 6.3 | — |

*Stabilized inverse-probability-of-treatment-weighted (IPTW) 3-year mortality risk differences (RD; treated − comparator) within diagnostic-time risk groups, in the pooled (training + validation) fixed-horizon-evaluable cohorts, with post-weighting balance and weight diagnostics. “Residual SMD (max)” is the largest absolute standardized mean difference across covariates after weighting; “Max stab. weight” is the maximum stabilized weight; the flag marks groups with residual imbalance (max |SMD| >0.1). “Any local therapy” = surgery and/or radiation. Comparator = SEER “documented yes” versus “no/unknown” (not confirmed untreated); SEER does not capture androgen-deprivation therapy or androgen-receptor pathway inhibitors. All estimates are observational and hypothesis-generating; the corresponding risk differences are visualized in Supplementary Figure S1.*

## Table S5. Survival-modeling sensitivity analyses (diagnostic-time predictors)

| Model | Endpoint | Harrell C-index (95% CI) | 3-year AUC (95% CI) | 3-year Brier (95% CI) |
| --- | --- | --- | --- | --- |
| Cox proportional hazards | OM | 0.656 (0.651–0.661) | 0.698 (0.691–0.706) | 0.234 (0.231–0.238) |
| Cause-specific Cox | CSM | 0.653 (not reported¹) | 0.694 (0.687–0.702) | 0.236 (0.232–0.240) |
| Fine–Gray competing-risk | CSM | 0.639 (0.637–0.648) | 0.676 (0.668–0.684) | 0.230 (0.229–0.231) |
| Random survival forest | OM | 0.650 (0.645–0.655) | 0.700 (0.693–0.707) | 0.222 (0.220–0.224) |
| Random survival forest | CSM | 0.647 (0.642–0.652) | 0.695 (0.687–0.703) | 0.221 (0.220–0.223) |

*¹ The confidence interval for the cause-specific Cox C-index is not reported owing to instability of the bootstrap estimate for this model. All models gave broadly consistent moderate discrimination and did not outperform the primary fixed-time CatBoost model.*

## Table S6. Additional robustness analyses (discrimination)

| Analysis | CSM n | CSM AUC | OM n | OM AUC |
| --- | --- | --- | --- | --- |
| Primary model (full temporal validation; reference) | 16,806 | 0.701 | 19,122 | 0.704 |
| 0-month survivors retained | 17,290 | 0.711 | 19,801 | 0.715 |
| 3-month landmark (survivors ≥3 months) | 16,146 | 0.687 | 18,214 | 0.690 |
| 6-month landmark (survivors ≥6 months) | 15,434 | 0.672 | 17,264 | 0.677 |
| Complete cases (PSA and Gleason known) | 9,190 | 0.685 | 10,104 | 0.682 |
| Stress subgroup: age ≥85 years | 2,277 | 0.630 | 2,874 | 0.628 |
| Stress subgroup: liver metastasis | 720 | 0.623 | 814 | 0.614 |
| Stress subgroup: brain metastasis | 159 | 0.621 | 191 | 0.632 |

*Area under the ROC curve for the primary diagnostic-time CatBoost model under prespecified robustness analyses. Retaining 0-month survivors slightly increased discrimination, and landmark and complete-case analyses were concordant with the primary result. Discrimination fell in the smallest, most information-limited stress subgroups (oldest patients; liver or brain metastasis). A strict treatment-coding sensitivity analysis (documented-yes vs strictly-no) was also concordant (not tabulated). Full prespecified subgroup and missingness analyses are detailed in Tables S12 and S13.*

## Table S7. SEER-documented treatment by diagnostic-time risk group (observational)

| Evaluable cohort | Risk group | n | Chemotherapy n (%) | Radiation n (%) | Surgery n (%) | Any local therapy n (%) |
| --- | --- | --- | --- | --- | --- | --- |
| Cancer-specific | Low | 12,817 | 2,338 (18.2) | 3,521 (27.5) | 1,762 (13.7) | 4,818 (37.6) |
| Cancer-specific | Intermediate | 12,209 | 2,334 (19.1) | 3,137 (25.7) | 1,135 (9.3) | 4,035 (33.0) |
| Cancer-specific | High | 10,785 | 1,062 (9.8) | 2,031 (18.8) | 886 (8.2) | 2,769 (25.7) |
| Cancer-specific | All | 35,811 | 5,734 (16.0) | 8,689 (24.3) | 3,783 (10.6) | 11,622 (32.5) |
| Overall | Low | 14,384 | 2,676 (18.6) | 3,945 (27.4) | 1,911 (13.3) | 5,361 (37.3) |
| Overall | Intermediate | 13,776 | 2,372 (17.2) | 3,383 (24.6) | 1,236 (9.0) | 4,367 (31.7) |
| Overall | High | 12,324 | 1,047 (8.5) | 2,180 (17.7) | 1,069 (8.7) | 3,079 (25.0) |
| Overall | All | 40,484 | 6,095 (15.1) | 9,508 (23.5) | 4,216 (10.4) | 12,807 (31.6) |

*Documented treatment within diagnostic-time risk groups in the pooled (training + validation) fixed-horizon-evaluable cohorts; percentages are within risk group. “Any local therapy” = surgery and/or radiation. Treatment is SEER “documented yes” versus “no/unknown,” and SEER does not capture androgen-deprivation therapy or androgen-receptor pathway inhibitors. The overall rates are consistent with the full-cohort rates in Table 1 (chemotherapy 15.0%, radiation 23.5%, surgery 10.4%). Documented local and systemic therapy was less frequent in the highest-risk group—plausibly reflecting frailty, competing priorities, or under-capture—but these distributions are descriptive and observational only.*

## Table S8. TRIPOD+AI reporting checklist

Completed for this study following the TRIPOD+AI statement (Collins GS, et al. BMJ 2024;385:e078378). Items are grouped by the checklist’s reporting domains; locations refer to the main manuscript unless a Supplementary (S) item is indicated. All items were addressed.

| # | Reporting domain — item | Reported in |
| --- | --- | --- |
| 1 | **Title** — identifies the study as development and validation of a multivariable prediction model, the target population, and the predicted outcomes | Title |
| 2 | **Abstract** — structured summary of objectives, data, methods, results, and conclusions | Abstract |
| 3 | **Introduction: background** — clinical context, rationale, and intended use; reference to existing models | §1 |
| 4 | **Introduction: objectives** — study objectives (development and temporal validation) | §1 |
| 5 | **Data source** — source(s) for development and validation and the rationale | §2.1–§2.2; §2.7; Suppl. Methods |
| 6 | **Study dates** — accrual and follow-up window (diagnosis 2010–2020) | §2.1–§2.3; §2.7 |
| 7 | **Setting** — care setting, number and location of registries | §2.1 (SEER 17 registries); §2.7 (single Chinese tertiary centre) |
| 8 | **Eligibility criteria** — participant inclusion/exclusion and case selection | §2.1; §2.7; Suppl. Methods; Table S9 |
| 9 | **Treatments received** — documented treatments and their handling | §2.8; Table 1; Tables S4, S7 |
| 10 | **Outcome** — definition, fixed-horizon evaluability rule, and assessment | §2.3 |
| 11 | **Predictors** — definition, timing (diagnostic-time only), and measurement | §2.4; Table S9 |
| 12 | **Sample size** — derivation of the analytic cohort and validation sets | §2.1; §2.5; §3.1; Figure 1 |
| 13 | **Missing data** — handling (unknown retained as explicit categories; sensitivity analyses) | §2.4; §2.10; Table S13 |
| 14 | **Data preparation** — preprocessing and one-hot encoding (identical feature space) | §2.5 |
| 15 | **Model — type and development** — seven algorithms; primary CatBoost; training procedure | §2.5 |
| 16 | **Model — hyperparameter tuning** — 20-candidate, 5-fold cross-validation | §2.5 |
| 17 | **Model output** — predicted 3-year mortality probability | §2.3; §2.6 |
| 18 | **Performance measures** — discrimination, calibration, overall performance, decision-curve utility | §2.6 |
| 19 | **Model evaluation** — temporal validation, independent external validation in a non-SEER cohort, and temporal internal–external cross-validation; IPCW fixed-time check | §2.2; §2.6; §2.7; §3.9; §3.10; Tables S10, S22, S23 |
| 20 | **Risk groups** — definition by training-cohort risk tertiles | §2.6; §3.5 |
| 21 | **Class imbalance** — outcome prevalence high (CSM ~46%, OM ~53%); no resampling applied | Not applicable (no resampling) |
| 22 | **Fairness / subgroup performance** — prespecified subgroup discrimination and calibration | §2.10; §3.8; Table S12 |
| 23 | **Interpretability / explainability** — full validation-set SHAP | §2.6; §3.4; Figure 4 |
| 24 | **Open science: funding** | Funding |
| 25 | **Open science: conflicts of interest** | Conflict of Interest |
| 26 | **Open science: protocol/registration** — analyses prespecified in Methods; no separate registration | §2 (prespecified) |
| 27 | **Open science: data availability** — SEER data-use agreement; not redistributable | Data Availability |
| 28 | **Open science: code availability** — public reproducibility repository (Zenodo) | Data Availability |
| 29 | **Open science: use of generative AI** — disclosed, scoped to drafting/language editing | Acknowledgements |
| 30 | **Results: participants** — flow and baseline characteristics | §3.1; Table 1; Figure 1 |
| 31 | **Results: model specification** — discrimination/calibration of all algorithms | §3.2–§3.3; Table 2; Table S1 |
| 32 | **Results: model performance** — discrimination, calibration, clinical utility, IPCW, extended calibration | §3.2–§3.3; Figures 2–3; Tables S2, S10, S11 |
| 33 | **Results: model interpretation** — SHAP attributions and clinical plausibility | §3.4; Figure 4 |
| 34 | **Results: risk stratification & robustness** — risk-group gradient; survival and robustness sensitivity analyses | §3.5; §3.7–§3.8; Tables S5, S6, S12, S13 |
| 35 | **Discussion: interpretation** — findings and comparison with existing models | §4 |
| 36 | **Discussion: limitations** — information ceiling, missingness, preliminary single-centre external validation (attenuated discrimination, calibration drift), treatment-data gaps | §4 |
| 37 | **Discussion: implications and intended use** — risk communication and trial enrichment; not treatment selection | §4; §5 |

## Table S9. Analysis variable dictionary

| Analysis variable | Requested concept | SEER source field | Recoding / handling |
| --- | --- | --- | --- |
| Cohort: site/behavior | Malignant prostate cancer | Primary Site C61.9; malignant behavior; known age | SEER*Stat “Select Only” (Malignant Behavior, Known Age) + site filter (statement above) |
| Cohort: stage | Distant disease at diagnosis | Combined Summary Stage with Expanded Regional Codes (2004+) | = “Distant site(s)/node(s) involved” (statement above) |
| Cohort: year | 2010–2020 | Year of diagnosis | Confirms range; also temporal split (train 2010–2016 / validation 2017–2020) |
| Survival time | Survival ≥1 month | Survival months | Main cohort retains ≥1 month; 0/Unknown only in sensitivity analysis |
| Age | Age at diagnosis | Age recode (incl. 90+) | SEER age-group midpoint (continuous) |
| Year of diagnosis | Year | Year of diagnosis | Continuous diagnosis-time predictor |
| Race | Race | Race recode (White/Black/API/AIAN) | Unknown retained as an explicit category |
| Marital status | Marital status | Marital status at diagnosis | Unknown retained as an explicit category |
| PSA | PSA | PSA lab-value recode (2010+) | Categorized; ≥98 ng/mL is a valid ceiling top-code, not missing (46.8% of the cohort, so PSA is effectively dichotomised at the top of its range and is best read as a coarse ordinal marker); unknown retained |
| Gleason | Gleason score | Gleason clinical recode (2010+) | Categorized; “not assessed/no biopsy” and other information-limited values retained |
| Bone metastasis | Bone metastasis | SEER combined mets at DX – bone (2010+) | Yes/No/Unknown retained |
| Liver metastasis | Liver metastasis | SEER combined mets at DX – liver (2010+) | Yes/No/Unknown retained |
| Lung metastasis | Lung metastasis | SEER combined mets at DX – lung (2010+) | Yes/No/Unknown retained |
| Brain metastasis | Brain metastasis | SEER combined mets at DX – brain (2010+) | Yes/No/Unknown retained |
| OM endpoint | 3-year overall mortality | Vital status + survival months | Event = death ≤36 mo; non-event = survival ≥36 mo; censored <36 mo not evaluable |
| CSM endpoint | 3-year cancer-specific mortality | Cause-specific death classification + survival months | Event = cancer death ≤36 mo; non-event = survival ≥36 mo; other-cause death or censoring <36 mo not evaluable |
| Surgery (secondary) | Surgery | RX Summ–Surg Prim Site | 00 = none, 99 = unknown, 1–98 = documented; “no/unknown” ≠ confirmed untreated |
| Radiation (secondary) | Radiation | Radiation recode | Documented yes vs none/unknown; comparator not confirmed untreated |
| Chemotherapy (secondary) | Chemotherapy | Chemotherapy recode (yes, no/unk) | Documented yes vs no/unknown; comparator not confirmed untreated |

*Variables retained in the analytic export, with the requested concept, the SEER source field, and recoding/handling of unknown categories.*

## Table S10. IPCW fixed-time (36-month) validation of the primary model

| Endpoint | IPCW AUC | IPCW Brier | Complete-case AUC (reference) |
| --- | --- | --- | --- |
| Overall (all-cause) | 0.703 | 0.219 | 0.704 |
| Cancer-specific | 0.658 | 0.235 | 0.701 |

*Inverse-probability-of-censoring-weighted (IPCW) estimates on the entire validation cohort (n=19,473) at 36 months. Overall mortality uses all-cause death; cancer-specific mortality is the cause-specific cumulative/dynamic AUC with other-cause death as a competing event. For overall mortality the IPCW AUC (0.703) and Brier score closely matched the complete-case estimates (AUC 0.704), indicating that the overall-mortality estimand was robust to early censoring. For cancer-specific mortality the IPCW analysis targets a* ***different estimand*** *from the primary model—the absolute cumulative incidence of cancer death in the presence of competing other-cause mortality, rather than the complete-case net probability defined in Methods (Section 2.3). The lower cancer-specific IPCW AUC (0.658) and the predicted net risk exceeding the competing-risks cumulative incidence are therefore expected, reflecting the greater intrinsic difficulty of absolute competing-risk prediction rather than miscalibration of the primary net-risk model; this result is best read as directionally supportive of the model as a net-risk stratification tool rather than as confirmation of robustness to competing mortality.*

## Table S11. Extended calibration of the primary model

| Endpoint | Mean predicted | Mean observed | O:E ratio | Calibration-in-the-large (intercept) |
| --- | --- | --- | --- | --- |
| Cancer-specific | 0.501 | 0.458 | 0.91 | −0.19 |
| Overall | 0.558 | 0.526 | 0.94 | −0.15 |

*Calibration slope is in main-text Table 2 (1.12 cancer-specific, 1.08 overall). Decile observed-versus-predicted risk is shown in Figure S2. The model mildly over-predicts on average (O:E < 1).*

## Table S12. Subgroup discrimination and calibration in temporal validation

| Subgroup | CSM n | CSM events | CSM AUC | CSM Brier | CSM slope | OM n | OM events | OM AUC | OM Brier | OM slope |
| --- | --- | --- | --- | --- | --- | --- | --- | --- | --- | --- |
| Age <70 | 7,395 | 2,804 | 0.676 | 0.216 | 1.15 | 7,973 | 3,401 | 0.673 | 0.224 | 1.10 |
| Age 70–79 | 5,054 | 2,214 | 0.683 | 0.223 | 1.22 | 5,807 | 2,979 | 0.676 | 0.227 | 1.14 |
| Age ≥80 | 4,357 | 2,672 | 0.648 | 0.223 | 1.09 | 5,342 | 3,668 | 0.645 | 0.203 | 1.03 |
| Race: White | 12,756 | 5,865 | 0.698 | 0.222 | 1.10 | 14,443 | 7,586 | 0.704 | 0.220 | 1.07 |
| Race: Black | 2,693 | 1,295 | 0.711 | 0.217 | 1.29 | 3,133 | 1,741 | 0.707 | 0.216 | 1.20 |
| Race: Asian/Pacific Islander | 1,140 | 454 | 0.712 | 0.210 | 1.20 | 1,304 | 620 | 0.715 | 0.216 | 1.18 |
| Race: American Indian/Alaska Native | 123 | 55 | 0.608 | 0.242 | 0.67 | 133 | 65 | 0.622 | 0.242 | 0.66 |
| Race: Unknown | 94 | 21 | 0.716 | 0.170 | 0.89 | 109 | 36 | 0.721 | 0.196 | 0.99 |
| PSA known | 14,885 | 6,623 | 0.700 | 0.220 | 1.12 | 16,845 | 8,617 | 0.703 | 0.220 | 1.08 |
| PSA unknown | 1,921 | 1,067 | 0.685 | 0.222 | 1.03 | 2,277 | 1,431 | 0.685 | 0.211 | 1.00 |
| Gleason known | 9,843 | 3,576 | 0.685 | 0.214 | 1.08 | 10,851 | 4,608 | 0.682 | 0.224 | 1.02 |
| Gleason unknown | 6,963 | 4,114 | 0.633 | 0.229 | 0.91 | 8,271 | 5,440 | 0.642 | 0.213 | 0.91 |
| Non-visceral metastasis | 14,645 | 6,392 | 0.692 | 0.221 | 1.14 | 16,683 | 8,470 | 0.699 | 0.221 | 1.11 |
| Visceral metastasis | 2,161 | 1,298 | 0.696 | 0.213 | 0.98 | 2,439 | 1,578 | 0.691 | 0.205 | 0.91 |
| Diagnosis year 2017 | 3,863 | 1,808 | 0.693 | 0.222 | 1.04 | 4,359 | 2,313 | 0.698 | 0.220 | 1.01 |
| Diagnosis year 2018 | 4,217 | 1,936 | 0.691 | 0.222 | 1.04 | 4,728 | 2,458 | 0.691 | 0.223 | 0.99 |
| Diagnosis year 2019 | 4,373 | 1,969 | 0.703 | 0.219 | 1.16 | 5,039 | 2,646 | 0.708 | 0.218 | 1.12 |
| Diagnosis year 2020 | 4,353 | 1,977 | 0.714 | 0.217 | 1.23 | 4,996 | 2,631 | 0.718 | 0.215 | 1.19 |

*Per-subgroup n, events (3-year deaths), AUC, Brier score, and calibration slope in the temporal validation cohort. Discrimination is lowest where prognostic information is limited (unknown Gleason; the oldest patients) and in the very small American Indian/Alaska Native subgroup; calibration is stable across most strata.*

## Table S13. Missingness sensitivity (handling of unknown PSA and Gleason)

| Strategy | CSM n | CSM events | CSM AUC | CSM Brier | OM n | OM events | OM AUC | OM Brier |
| --- | --- | --- | --- | --- | --- | --- | --- | --- |
| Missing-indicator (primary; unknown retained as a category) | 16,806 | 7,690 | 0.701 | 0.220 | 19,122 | 10,048 | 0.704 | 0.219 |
| Complete-case (PSA and Gleason known) | 9,190 | 3,286 | 0.685 | 0.213 | 10,104 | 4,222 | 0.682 | 0.223 |
| Simple imputation (mode) | 16,806 | 7,690 | 0.697 | 0.221 | 19,122 | 10,048 | 0.700 | 0.220 |

*Retaining unknown values as explicit categories gave the best discrimination while preserving the full sample, supporting the primary approach. Complete-case analysis restricts to the subset with both PSA and Gleason known and therefore has fewer patients and events.*

*Tables S10–S13 and Figure S2 were generated with the deposited reproducibility pipeline using the same CV-tuned primary CatBoost model as the main analysis (reproducing the reported validation AUC 0.701/0.704 and calibration slope 1.12/1.08).*

## Table S14. Comparison with prior prognostic models in metastatic prostate cancer

| Study | Population; data source | Sample (n) | Validation design | Discrimination (as reported) | Calibration / decision curve | Independent external validation | Open code |
| --- | --- | --- | --- | --- | --- | --- | --- |
| Halabi 2014 [15] | mCRPC, first-line chemotherapy; phase III trials | 1,050 | Train/test split + independent trial | Time-dependent AUC for overall survival, as reported (not directly comparable) | Calibration: yes; DCA: no | Yes (independent trial) | No |
| Hou 2019 [16] | Bone-metastatic PCa; SEER | 1,556 (+711) | SEER subset split by diagnosis era | 3-year cancer-specific survival AUC 0.70 (as reported) | Calibration: yes; DCA: no | No (within-SEER subset only) | No |
| Sun 2022 [34] | Bone-metastatic PCa; SEER (2010–2016) | 7,315 | Internal 7:3 split | C-index 0.71 (cancer-specific survival), 0.70 (overall survival), as reported | Calibration: yes; DCA: yes | No | No |
| This study | De novo metastatic PCa; SEER 17 (2010–2020) | 41,057 | Temporal split (train 2010–2016, validation 2017–2020) | 3-year AUC 0.70 (temporal validation) | Calibration: yes; DCA: yes | Yes (independent non-SEER single-centre cohort; preliminary, AUC ≈0.59; Table S23) | Yes (Zenodo) |

*The three prior models predict cancer-specific and/or overall survival and the present model predicts the corresponding 3-year mortality, with broadly comparable discrimination (C-index/AUC about 0.70) on their internal or trial-based evaluation. The distinguishing features of the present work are its de novo metastatic (hormone-sensitive) setting and large national sample, a temporally independent validation set rather than a random split, explicit calibration and decision-curve assessment, full model interpretability (SHAP, Figure 4), and openly released code and model objects. Hou et al. described a within-SEER subset diagnosed in different years as external validation; that design corresponds to our temporal validation, and we additionally provide a preliminary independent external validation in a non-SEER single-centre cohort (Table S23). mCRPC, metastatic castration-resistant prostate cancer; CSM, cancer-specific mortality; OM, overall mortality; AUC, area under the receiver-operating-characteristic curve; DCA, decision-curve analysis.*

## Table S15. Missingness sensitivity: multiple imputation (MAR) and pattern-mixture (MNAR)

| Endpoint | Sensitivity analysis | Validation AUC | Calibration slope | Low-risk rate | Int-risk rate | High-risk rate |
| --- | --- | --- | --- | --- | --- | --- |
| CSM | Missing-indicator (primary; reference) | 0.701 | 1.12 | 0.281 | 0.492 | 0.669 |
| CSM | Multiple imputation, MAR (m=10), pooled | 0.681 (0.678–0.683) | 1.04 | 0.303 | 0.480 | 0.647 |
| CSM | Pattern-mixture, MNAR (Gleason +0.5 logit) | 0.684 | 1.05 | 0.301 | 0.482 | 0.649 |
| CSM | Pattern-mixture, MNAR (+1.0) | 0.687 | 1.05 | 0.295 | 0.487 | 0.658 |
| CSM | Pattern-mixture, MNAR (+1.5) | 0.690 | 1.06 | 0.291 | 0.490 | 0.657 |
| CSM | Pattern-mixture, MNAR (+2.0) | 0.693 | 1.07 | 0.287 | 0.490 | 0.661 |
| OM | Missing-indicator (primary; reference) | 0.704 | 1.08 | 0.337 | 0.566 | 0.738 |
| OM | Multiple imputation, MAR (m=10), pooled | 0.684 (0.682–0.686) | 1.00 | 0.364 | 0.554 | 0.716 |
| OM | Pattern-mixture, MNAR (Gleason +0.5 logit) | 0.686 | 1.02 | 0.363 | 0.554 | 0.722 |
| OM | Pattern-mixture, MNAR (+1.0) | 0.690 | 1.02 | 0.358 | 0.556 | 0.724 |
| OM | Pattern-mixture, MNAR (+1.5) | 0.693 | 1.03 | 0.353 | 0.558 | 0.724 |
| OM | Pattern-mixture, MNAR (+2.0) | 0.695 | 1.03 | 0.349 | 0.561 | 0.725 |

*Sensitivity of the primary diagnostic-time CatBoost model to the handling of unknown PSA and Gleason. Multiple imputation (MAR) multiply imputes the unknown categories (m=10) from the observed diagnostic-time predictors and refits the primary model on each completed dataset; the pooled validation AUC (about 0.68) reproduces the complete-case result (Table S13) and falls below the missing-indicator primary, indicating that the unknown categories carry prognostic information. The pattern-mixture (MNAR) analysis progressively shifts the imputed Gleason of the unknown rows toward grade 9-10 by a log-odds delta; discrimination and the risk-group gradient remain stable (indeed slightly higher) across this range, showing the conclusions are robust to plausible departures from missing-at-random. Risk-group rates are observed 3-year event rates by training-tertile group. m, number of imputations; MAR, missing-at-random; MNAR, missing-not-at-random.*

## Table S16. Histology composition and adenocarcinoma-only sensitivity analysis

| Endpoint | Cohort | Validation n (events) | Validation AUC | Calibration slope | Low-risk rate | Int-risk rate | High-risk rate |
| --- | --- | --- | --- | --- | --- | --- | --- |
| CSM | Full cohort (all histologies) | 16,806 (7,690) | 0.701 | 1.12 | 0.281 | 0.492 | 0.669 |
| CSM | Adenocarcinoma only | 14,673 (6,197) | 0.687 | 1.10 | 0.254 | 0.455 | 0.609 |
| OM | Full cohort (all histologies) | 19,122 (10,048) | 0.704 | 1.08 | 0.337 | 0.566 | 0.738 |
| OM | Adenocarcinoma only | 16,511 (8,074) | 0.688 | 1.04 | 0.318 | 0.519 | 0.678 |

*Histology composition of the main cohort (n=41,057): adenocarcinoma 85.6%; carcinoma or malignant neoplasm not otherwise specified 13.0%; small-cell or neuroendocrine carcinoma 1.2%; other specified non-adenocarcinoma 0.3%. The adenocarcinoma-only sensitivity refits and re-validates the primary diagnostic-time CatBoost model in the adenocarcinoma subset only; discrimination is modestly lower than in the full cohort while calibration is maintained, indicating the primary results are not driven by the small fraction of rare aggressive variants. Risk-group rates are observed 3-year event rates by training-tertile group; events in parentheses. Histology was obtained by re-extracting the same case selection with the Histologic Type ICD-O-3 variable added.*

## Table S17. Characteristics of patients with assessed versus not-assessed Gleason score (informative missingness)

| Characteristic | Gleason assessed (n=23,960) | Gleason not assessed/no biopsy (n=17,097) | SMD |
| --- | --- | --- | --- |
| n (% of cohort) | 23,960 (58.4%) | 17,097 (41.6%) |  |
| Age, mean (years) | 69.9 | 74.7 | 0.44 |
| Year of diagnosis, mean | 2015.7 | 2016.0 | 0.08 |
| PSA >=98 ng/mL (ceiling) | 40.3% | 55.8% | 0.31 |
| PSA unknown | 7.4% | 19.7% | 0.36 |
| Bone metastasis | 86.2% | 86.7% | 0.02 |
| Liver metastasis | 3.3% | 5.4% | 0.10 |
| Lung metastasis | 6.9% | 10.6% | 0.13 |
| Brain metastasis | 0.6% | 1.7% | 0.10 |
| Chemotherapy (documented) | 16.5% | 13.0% | -0.10 |
| Radiation (documented) | 24.6% | 22.0% | -0.06 |
| Surgery (documented) | 15.4% | 3.4% | -0.41 |
| 3-year cancer-specific mortality | 41.4% | 61.3% | 0.40 |
| 3-year overall mortality | 47.0% | 67.3% | 0.41 |

*Comparison of the 23,960 patients with an assessed Gleason score and the 17,097 with Gleason not assessed/no biopsy across diagnostic-time covariates, SEER-documented treatment, and observed 3-year mortality. Patients without an assessed Gleason were older, had higher PSA and more visceral metastasis, were far less likely to have documented surgery, and had substantially higher 3-year mortality, indicating that the not-assessed/no-biopsy category is informative (missing-not-at-random) and marks advanced, frail, or incompletely worked-up disease rather than a transportable tumour-biology variable. Because the meaning of an absent biopsy depends on local diagnostic and referral practice, a model that uses this category should be recalibrated before use in other settings. SMD, standardized mean difference (not-assessed minus assessed); documented treatment denotes a SEER documented-yes code; mortality is the observed 3-year event rate among the fixed-horizon-evaluable patients in each group.*

## Table S18. Estimands: overall, net cancer-specific, and competing-risks cumulative-incidence quantities

| Quantity | What it estimates | How obtained | Interpretation |
| --- | --- | --- | --- |
| Overall mortality (OM; primary) | Probability of death from any cause by 36 months | Complete-case among patients with determinate 36-month vital status | Absolute all-cause mortality risk; directly interpretable |
| Cancer-specific net risk (CSM; primary) | Cause-specific (net) probability of cancer death by 36 months, removing other-cause deaths | Complete-case; other-cause death before 36 months is non-evaluable | A net-risk score for cancer-specific stratification; NOT the absolute probability of cancer death in the presence of competing mortality |
| Competing-risks cumulative incidence (sensitivity) | Absolute cumulative incidence of cancer death with other-cause death as a competing event | Fine-Gray subdistribution model and IPCW on the full cohort | Absolute cancer-death incidence accounting for competing mortality; intrinsically harder to predict |

*The primary models target the first two quantities. The cancer-specific (net) target is appropriate for risk stratification and matches the diagnostic-time classification setting, but it is not the absolute competing-risks cumulative incidence; the third quantity is examined as a sensitivity analysis (Tables S5 and S10) and, as expected for absolute competing-risk prediction, shows lower discrimination. OM, overall mortality; CSM, cancer-specific mortality; IPCW, inverse-probability-of-censoring weighting.*

## Table S19. Final hyperparameters of the primary diagnostic-time CatBoost models

| Hyperparameter | Cancer-specific (CSM) | Overall (OM) |
| --- | --- | --- |
| Tree depth | 4 | 4 |
| Learning rate | 0.05 | 0.05 |
| Iterations (trees) | 200 | 300 |
| L2 leaf regularization | 5 | 5 |
| One-hot feature count | 35 | 35 |
| Loss function | Logloss | Logloss |
| Random seed | 20260529 | 20260529 |

*Final locked hyperparameters of the primary diagnostic-time CatBoost models, selected by 20-candidate, 5-fold cross-validation within the training cohort (the iteration grid was capped to keep the AUC-scored search well-calibrated). CatBoost was trained on the shared one-hot feature space. The model objects (primary_catboost_{css,os}.cbm) and a JSON snapshot recording these values, the feature list, the tree count, and a SHA-256 checksum are deposited.*

## Table S20. SHAP importance aggregated to the original diagnostic-time predictors

| Predictor | Cancer-specific (% of total \|SHAP\|) | Overall (% of total \|SHAP\|) |
| --- | --- | --- |
| Gleason score | 28.6 | 25.6 |
| Age | 21.2 | 23.8 |
| PSA | 12.9 | 13.0 |
| Year of diagnosis | 9.4 | 9.2 |
| Liver metastasis | 8.0 | 7.4 |
| Bone metastasis | 6.2 | 5.2 |
| Marital status | 6.0 | 8.4 |
| Race | 4.1 | 4.1 |
| Lung metastasis | 2.2 | 2.0 |
| Brain metastasis | 1.6 | 1.4 |

*Mean absolute SHAP value summed across the one-hot columns belonging to each original diagnostic-time predictor, as a percentage of the total, in the temporal validation cohort. This aggregates the per-category contributions shown in the Figure 4 beeswarm to the level of the original variables; Gleason score (including the not-assessed category) and age are the dominant predictors.*

## Table S21. Decision-curve net benefit at selected threshold probabilities

| Endpoint | Threshold probability | CatBoost (primary) | Logistic regression | Treat all | Treat none |
| --- | --- | --- | --- | --- | --- |
| CSM | 20% | 0.326 | 0.325 | 0.322 | 0.000 |
| CSM | 30% | 0.249 | 0.245 | 0.225 | 0.000 |
| CSM | 50% | 0.101 | 0.082 | -0.085 | 0.000 |
| CSM | 70% | 0.017 | 0.011 | -0.808 | 0.000 |
| OM | 20% | 0.409 | 0.408 | 0.407 | 0.000 |
| OM | 30% | 0.335 | 0.330 | 0.322 | 0.000 |
| OM | 50% | 0.171 | 0.156 | 0.051 | 0.000 |
| OM | 70% | 0.042 | 0.031 | -0.582 | 0.000 |

*Decision-curve net benefit at selected threshold probabilities in the temporal validation cohort (full curves in Figure 3E,F). At low thresholds, where intervening on almost everyone is reasonable in this high-mortality disease, the model net benefit is close to treat-all; across the clinically relevant 30-70% range it exceeds both treat-all (which becomes negative) and treat-none, quantifying the advantage visible in Figure 3.*

## Table S22. Per-year denominators and temporal internal-external cross-validation performance (leave-one-calendar-year-out)

| Endpoint | Diagnosis year | N evaluable | Events | Event rate | AUC (95% CI) | Calibration slope | CITL | O:E | Brier |
| --- | --- | --- | --- | --- | --- | --- | --- | --- | --- |
| Net cancer-specific (CSM) | 2010 | 2147 | 1201 | 0.559 | 0.703 (0.678–0.724) | 1.073 | 0.099 | 1.040 | 0.216 |
|  | 2011 | 2235 | 1203 | 0.538 | 0.678 (0.656–0.700) | 0.905 | -0.051 | 0.980 | 0.225 |
|  | 2012 | 2428 | 1296 | 0.534 | 0.692 (0.673–0.713) | 1.029 | -0.084 | 0.967 | 0.221 |
|  | 2013 | 2640 | 1472 | 0.558 | 0.670 (0.652–0.690) | 0.894 | 0.055 | 1.022 | 0.225 |
|  | 2014 | 2819 | 1459 | 0.518 | 0.688 (0.668–0.706) | 0.994 | -0.099 | 0.959 | 0.223 |
|  | 2015 | 3222 | 1664 | 0.516 | 0.707 (0.689–0.725) | 1.142 | -0.047 | 0.980 | 0.216 |
|  | 2016 | 3514 | 1722 | 0.490 | 0.700 (0.683–0.717) | 1.054 | -0.075 | 0.967 | 0.219 |
|  | 2017 | 3863 | 1808 | 0.468 | 0.693 (0.675–0.708) | 0.954 | -0.114 | 0.949 | 0.222 |
|  | 2018 | 4217 | 1936 | 0.459 | 0.698 (0.683–0.713) | 0.983 | -0.046 | 0.979 | 0.219 |
|  | 2019 | 4373 | 1969 | 0.450 | 0.711 (0.694–0.725) | 1.052 | -0.018 | 0.992 | 0.215 |
|  | 2020 | 4353 | 1977 | 0.454 | 0.716 (0.701–0.731) | 1.098 | -0.007 | 0.997 | 0.213 |
| Net cancer-specific (CSM) | **Pooled (patient-once)** | 35811 | 17707 | 0.494 | **0.701 (0.696–0.707)** | 1.019 | -0.039 | 0.983 | 0.219 |
| Net cancer-specific (CSM) | **Random-effects summary** | – | – | – | **0.697 (0.689–0.705)** | – | – | – | – |
| Overall (OM) | 2010 | 2410 | 1466 | 0.608 | 0.698 (0.678–0.718) | 1.054 | 0.081 | 1.030 | 0.211 |
|  | 2011 | 2526 | 1498 | 0.593 | 0.682 (0.662–0.703) | 0.939 | -0.023 | 0.992 | 0.217 |
|  | 2012 | 2754 | 1631 | 0.592 | 0.698 (0.679–0.719) | 1.075 | -0.058 | 0.980 | 0.213 |
|  | 2013 | 2969 | 1811 | 0.610 | 0.675 (0.655–0.695) | 0.919 | 0.063 | 1.022 | 0.217 |
|  | 2014 | 3167 | 1810 | 0.572 | 0.689 (0.670–0.708) | 1.010 | -0.098 | 0.964 | 0.219 |
|  | 2015 | 3603 | 2048 | 0.568 | 0.711 (0.696–0.729) | 1.164 | -0.044 | 0.983 | 0.212 |
|  | 2016 | 3933 | 2147 | 0.546 | 0.699 (0.682–0.715) | 1.049 | -0.062 | 0.976 | 0.218 |
|  | 2017 | 4359 | 2313 | 0.531 | 0.700 (0.685–0.715) | 0.998 | -0.083 | 0.967 | 0.219 |
|  | 2018 | 4728 | 2458 | 0.520 | 0.696 (0.682–0.711) | 0.975 | -0.060 | 0.976 | 0.220 |
|  | 2019 | 5039 | 2646 | 0.525 | 0.716 (0.701–0.731) | 1.093 | 0.008 | 1.003 | 0.215 |
|  | 2020 | 4996 | 2631 | 0.527 | 0.720 (0.706–0.734) | 1.094 | -0.006 | 0.997 | 0.213 |
| Overall (OM) | **Pooled (patient-once)** | 40484 | 22459 | 0.555 | **0.703 (0.698–0.708)** | 1.037 | -0.030 | 0.989 | 0.216 |
| Overall (OM) | **Random-effects summary** | – | – | – | **0.699 (0.691–0.707)** | – | – | – | – |

*Leave-one-calendar-year-out internal-external cross-validation within SEER (temporal, not geographic): each diagnosis year 2010–2020 is held out once and scored by the frozen primary CatBoost model refit on all other years (refit-not-retune); every patient is predicted exactly once. All 11 folds met the pre-specified Tier-A threshold (≥200 patients and ≥100 events). CSM is a* ***net (cause-specific)*** *3-year mortality probability — patients dying of competing causes before 36 months are removed rather than treated as event-free (see Table S18); the evaluable fraction therefore drifts downward over calendar time (CSM 88.6% in 2010 to 84.7% in 2020; OM 99.4% to 97.2%). year_dx is retained as a model feature, so the held-out interior year is interpolated. The random-effects summary is a DerSimonian–Laird pooling on the logit-AUC scale; its 95% prediction interval — the transportability statement — is 0.672–0.721 (CSM; I² 54%, τ² 0.0022) and 0.673–0.725 (OM; I² 61%, τ² 0.0025). Because leave-one-year folds share most of their training data, the pooled patient-level CI is anti-conservative; the year-cluster bootstrap CI (CSM 0.693–0.708; OM 0.694–0.710) and the prediction interval are the transportability intervals. AUC, area under the ROC curve; CITL, calibration-in-the-large; O:E, observed-to-expected ratio. Full reproduction in the deposited s13b_temporal_iecv.py.*

## Table S23. External validation in an independent, non-SEER single-centre cohort (locked models applied without modification)

**(a) Discrimination, calibration, and overall performance**

| Endpoint | n (events) | AUC (95% CI) | Calibration slope | CITL intercept | O:E ratio | Brier |
| --- | --- | --- | --- | --- | --- | --- |
| 3-year CSM | 129 (65) | 0.59 (0.49–0.69) | 0.47 | +0.42 | 1.23 | 0.258 |
| 3-year OM | 146 (82) | 0.59 (0.50–0.68) | 0.47 | +0.40 | 1.19 | 0.255 |

**(b) Observed 3-year mortality by risk group, using the frozen training cut points**

| Endpoint | Risk group | n | Observed (external) | Observed (internal validation) |
| --- | --- | --- | --- | --- |
| CSM | Low | 88 | 44.3% | 28.1% |
| CSM | Intermediate | 25 | 64.0% | 49.2% |
| CSM | High | 16 | 62.5% | 66.9% |
| OM | Low | 99 | 50.5% | 33.7% |
| OM | Intermediate | 26 | 65.4% | 56.6% |
| OM | High | 21 | 71.4% | 73.8% |

*Independent cohort of 150 men with de novo metastatic prostate cancer from a single Chinese tertiary centre (The Second People’s Hospital of Wuhu; diagnosed 2011–2018), sharing no patients, registry, or coding system with SEER; 129 cancer-specific– and 146 overall-mortality–evaluable under the fixed 36-month rule. The locked primary CatBoost models were applied without any refitting or recalibration. AUC, area under the receiver-operating-characteristic curve (DeLong 95% CI); CITL, calibration-in-the-large intercept; O:E, observed-to-expected ratio. Risk groups use the frozen training-cohort risk tertiles (CSM 0.461/0.595; OM 0.515/0.657); internal-validation rates are the SEER temporal-validation event rates (Figure 5). Discrimination was attenuated relative to internal validation (≈0.70), with confidence intervals reaching ≈0.5 (no claim of significant external discrimination), and calibration drifted (slope <1, O:E >1, positive intercept). The risk-group ordering was preserved as a monotone low→intermediate→high gradient for overall mortality; for cancer-specific mortality the low group separated clearly from the higher groups, but the intermediate and high groups (n=25 and 16) did not separate from each other. As an internal control, the same scoring pipeline reproduced the locked internal validation AUC (0.701/0.704) and the risk-group cut points exactly. See Figure S4 for the external calibration curves and Table S24 for the case-mix comparison.*

## Table S24. Case-mix comparison: SEER temporal-validation cohort versus the independent external cohort

| Characteristic | SEER temporal validation (n=19,473) | External cohort (n=150) |
| --- | --- | --- |
| Age, median (years) | 72 | 72 |
| Age <70 / 70–79 / ≥80, % | 41.9 / 30.3 / 27.7 | 36.7 / 40.7 / 22.7 |
| Mapped to SEER “Asian or Pacific Islander” race code, % | 6.9 | 100 |
| PSA <10 / 10–19.9 / 20–97.9 / ≥98 / unknown, % | 8.4 / 8.6 / 24.1 / 47.0 / 12.0 | 2.0 / 8.7 / 47.3 / 42.0 / 0.0 |
| Gleason ≤6 / 7 / 8 / 9–10 / not assessed, % | 0.8 / 6.8 / 13.5 / 35.8 / 43.2 | 1.3 / 10.7 / 19.3 / 46.0 / 22.7 |
| Bone / liver / lung / brain metastasis (Yes), % | 86.5 / 4.3 / 9.3 / 1.0 | 86.7 / 7.3 / 6.0 / 2.7 |
| 3-year cancer-specific / overall mortality (evaluable), % | 45.8 / 52.5 | 50.4 / 56.2 |

*Column percentages; mortality is among patients evaluable under the fixed 36-month rule. The external cohort differs from the SEER development population in ways that plausibly compress achievable discrimination (a case-mix / spectrum effect) and may partly explain the attenuation, although genuine loss of transportability cannot be excluded on this small sample: PSA was concentrated in the 20–97.9 ng/mL band with essentially no missing values (0% vs 12%), and—most importantly—the Gleason “not assessed/no biopsy” category, one of the strongest contributors in the SHAP analysis (Figure 4), was far less prevalent externally (22.7% vs 43.2%), so the model could exploit less of its most discriminating diagnostic-time signal; the external cohort was also modestly higher-risk (3-year mortality ~4–5 percentage points higher). These differences are consistent with the attenuated external discrimination reported in Section 3.10 and Table S23.*

## Table S25. Model fit with and without year of diagnosis

| Endpoint | Model | n / events | AUC | AP | Brier | Calibration slope | Risk-group event rates (low / intermediate / high), % |
| --- | --- | --- | --- | --- | --- | --- | --- |
| CSM | With year of diagnosis (primary) | 16,806 / 7,690 | 0.701 | 0.647 | 0.220 | 1.115 | 28.1 / 49.2 / 66.9 |
| CSM | Without year of diagnosis | 16,806 / 7,690 | 0.701 | 0.647 | 0.224 | 1.130 | 24.9 / 46.1 / 64.4 |
| OM | With year of diagnosis (primary) | 19,122 / 10,048 | 0.704 | 0.707 | 0.219 | 1.079 | 33.7 / 56.6 / 73.8 |
| OM | Without year of diagnosis | 19,122 / 10,048 | 0.704 | 0.708 | 0.222 | 1.095 | 31.2 / 52.9 / 71.6 |

*Temporal validation cohort. Removing year of diagnosis leaves discrimination and average precision essentially unchanged but produces a small, consistent loss of overall fit and calibration and shifts the absolute risk-group event rates downward, indicating that year of diagnosis carries calendar-time information about declining baseline risk rather than adding rank-ordering signal. Because the rank ordering is preserved while the absolute level shifts, a year-of-diagnosis-free model would require re-derived risk-group cut points and local recalibration before use and is not a drop-in substitute. AP, average precision; CSM, cancer-specific mortality; OM, overall mortality.*

## Table S26. Absolute 36-month competing-risks cumulative incidence by diagnostic-time risk group

| Risk group | n | Cancer death, cumulative incidence, % (SE) | Other-cause death, cumulative incidence, % | All-cause mortality (1 - Kaplan-Meier), % |
| --- | --- | --- | --- | --- |
| Low | 7,173 | 25.7 (0.52) | 7.9 | 33.6 |
| Intermediate | 6,814 | 42.9 (0.60) | 12.2 | 55.1 |
| High | 5,486 | 54.7 (0.67) | 18.0 | 72.7 |
| Whole validation cohort | 19,473 | 39.9 (0.35) | 12.2 | 52.2 |

*Aalen-Johansen cumulative incidence at 36 months in the full temporal-validation cohort, with other-cause death treated as a competing event, tabulated by the diagnostic-time risk groups defined by the frozen training-cohort tertile cut points. This is a descriptive re-expression of the existing risk strata in absolute terms and is not a cumulative-incidence prediction model: the groups are formed from the primary classifier's predicted net risk and no new model is fitted. Two features are relevant to clinical interpretation: competing other-cause mortality rises steeply across the strata (7.9% to 18.0%), which is why the net cancer-specific quantity exceeds the absolute cancer-death probability in this elderly cohort; and the all-cause column (33.6 / 55.1 / 72.7%) is close to, without being identical to, the observed overall-mortality risk-group rates reported in Section 3.5 (33.7 / 56.6 / 73.8%), which are defined by the overall-mortality rather than the cancer-specific cut points. Reproduced by 01_R_scripts/14_cif_by_riskgroup.R.*

## Table S27. Comparison with a parsimonious three-variable clinical rule

| Endpoint | Model | AUC (95% CI) | Delta AUC vs CatBoost | DeLong p | Net benefit at 30% | Net benefit at 50% | Net benefit at 70% |
| --- | --- | --- | --- | --- | --- | --- | --- |
| CSM | CatBoost (primary) | 0.701 (0.693-0.708) | - | - | 0.249 | 0.101 | 0.017 |
| CSM | Logistic regression (35 columns) | 0.695 (0.686-0.703) | 0.006 | <0.001 | 0.245 | 0.082 | 0.011 |
| CSM | Three-variable rule (age, PSA, Gleason) | 0.671 (0.663-0.679) | 0.030 | <0.001 | 0.237 | 0.057 | 0.001 |
| OM | CatBoost (primary) | 0.704 (0.697-0.711) | - | - | 0.335 | 0.171 | 0.042 |
| OM | Logistic regression (35 columns) | 0.699 (0.692-0.707) | 0.005 | <0.001 | 0.330 | 0.156 | 0.031 |
| OM | Three-variable rule (age, PSA, Gleason) | 0.680 (0.673-0.687) | 0.024 | <0.001 | 0.326 | 0.139 | 0.019 |

*The three-variable rule is a logistic model containing only age, PSA category and Gleason category - the information a clinician can apply without software - fitted on the 2010-2016 training cohort and evaluated on the 2017-2020 temporal-validation cohort under the identical evaluability rule. The CatBoost and full 35-column logistic predictions are taken from the deposited validation-prediction files rather than re-fitted, so they reproduce the published values exactly. The primary model discriminates better than the parsimonious rule for both endpoints (Delta AUC 0.024-0.030; DeLong p<0.001) and the advantage in net benefit widens as the threshold rises, that is, in the range relevant to flagging higher-risk patients; the increment is nevertheless modest, consistent with the information ceiling of registry predictors discussed in Section 4. Net benefit is computed for the decision to flag a patient for intensified prognostic counselling, closer follow-up and trial referral. Reproduced by 00_full_pipeline_python/s14_three_variable_rule.py. CSM, cancer-specific mortality; OM, overall mortality.*

## Supplementary Note. Reusing the primary diagnostic-time model

Applying the locked primary models requires only the diagnostic-time predictors used in development: age (SEER age-group midpoint, continuous), year of diagnosis (continuous), race, marital status, PSA category, Gleason category, and bone, liver, lung, and brain metastasis categories (yes/no/unknown, as available). Categorical predictors are one-hot encoded exactly as in development, and unknown or not-assessed values are retained as their own explicit categories rather than imputed; the complete one-hot feature list and ordering are provided in the deposited predictor_dictionary/T4_onehot_feature_mapping.csv, which reproduces exactly the feature_names array recorded in the JSON snapshot deposited with each primary CatBoost model (the authoritative feature order consumed by the model).

The retrained endpoint-specific CatBoost models are deposited as outputs/models/primary_catboost_css.cbm and primary_catboost_os.cbm, each accompanied by a JSON snapshot recording the random seed, hyperparameters, feature list, and a SHA-256 checksum. A predicted 3-year mortality probability is obtained by loading the relevant model object and requesting its probability prediction for the one-hot feature vector assembled in the documented order.

Patients are assigned to diagnostic-time risk groups using cut points fixed at the tertiles of training-cohort predicted risk: cancer-specific 0.461217 and 0.595211, and overall 0.515143 and 0.657387 (predicted risk below the lower cut point = low risk, between the two cut points = intermediate, above the upper = high).

Because calibration can degrade outside the SEER development setting, any use in a different population should be preceded by local intercept-and-slope recalibration, with calibration and decision-curve net benefit re-assessed before deployment. The model is intended for diagnostic-time risk stratification and communication, not for treatment selection.

## Supplementary Figure S1. Risk-stratified treatment-associated mortality differences (observational)

Supplementary Figure S1

Stabilized-IPTW weighted risk differences (treated minus comparator) by treatment and risk group, for cancer-specific (A) and overall (B) 3-year mortality. Red indicates higher mortality with the documented treatment and blue lower; the value in each cell is the weighted risk difference. Open triangles mark cautious signals (residual imbalance or high/unstable weights) and asterisks mark weak/unstable signals. Observational association only: comparators are documented treatment versus no/unknown (not confirmed untreated), and SEER does not capture androgen-deprivation therapy or androgen-receptor pathway inhibitors. These analyses are hypothesis-generating and must not be interpreted as treatment effects.

## Supplementary Figure S2. Decile calibration of the primary model

Supplementary Figure S2

Mean predicted versus observed 3-year risk by decile of predicted risk in the temporal validation cohort, for cancer-specific and overall mortality. The dashed line denotes perfect calibration; points lie close to it with mild over-prediction, consistent with the calibration slopes (1.12/1.08) and observed-to-expected ratios (0.91/0.94) reported above.

## Supplementary Figure S3. Temporal internal-external cross-validation: per-year discrimination with random-effects pooling

Supplementary Figure S3

Forest plot of leave-one-calendar-year-out internal-external cross-validation within SEER (temporal, not geographic) for net cancer-specific (A) and overall (B) 3-year mortality. Each row is one held-out diagnosis year (2010–2020); the box marks the year’s AUC with area proportional to its inverse-variance weight and whiskers the 95% CI; the red diamond is the DerSimonian–Laird random-effects summary and the shaded band its 95% prediction interval (the transportability statement). Relative heterogeneity (I²) is annotated alongside the absolute per-year AUC range. Discrimination is stable across all 11 years (CSM 0.670–0.716; OM 0.675–0.720), with the prediction interval excluding any non-discriminating year.

## Supplementary Figure S4. External-cohort calibration of the primary models

Supplementary Figure S4

External validation in the independent, non-SEER single-centre cohort: mean predicted versus observed 3-year mortality for cancer-specific (A) and overall (B) death, with the locked models applied without any refitting or recalibration. Red points are quintiles of predicted risk (size proportional to group n) with Wilson 95% confidence intervals; the solid blue line is a logistic recalibration curve; the dashed line denotes perfect calibration. The recalibration curve is shallower than the diagonal (calibration slope 0.47) and lies above it at low predicted risk, reflecting under-prediction of absolute risk in this higher-mortality cohort (observed-to-expected ratios 1.23 and 1.19). CSM n=129; OM n=146.
